# miR24‐2 accelerates progression of liver cancer cells by activating Pim1 through tri‐methylation of Histone H3 on the ninth lysine

**DOI:** 10.1111/jcmm.15030

**Published:** 2020-02-06

**Authors:** Yuxin Yang, Shuting Song, Qiuyu Meng, Liyan Wang, Xiaonan Li, Sijie Xie, Yingjie Chen, Xiaoxue Jiang, Chen Wang, Yanan Lu, Xiaoru Xin, Hu Pu, Xin Gui, Tianming Li, Jie Xu, Jiao Li, Song Jia, Dongdong Lu

**Affiliations:** ^1^ Shanghai Putuo District People's Hospital School of Life Science and Technology Tongji University Shanghai China; ^2^ School of Medical Technology Shanghai University of Medicine and Health Sciences Shanghai China; ^3^ School of Medicine Tongji University Shanghai China

**Keywords:** H3K9me3, JMJD2A, liver cancer, miR24‐2, miR6079, Pim1

## Abstract

Several microRNAs are associated with carcinogenesis and tumour progression. Herein, our observations suggest both miR24‐2 and Pim1 are up‐regulated in human liver cancers, and miR24‐2 accelerates growth of liver cancer cells in vitro and in vivo. Mechanistically, miR24‐2 increases the expression of N6‐adenosine‐methyltransferase METTL3 and thereafter promotes the expression of miR6079 via RNA methylation modification. Furthermore, miR6079 targets JMJD2A and then increased the tri‐methylation of histone H3 on the ninth lysine (H3K9me3). Therefore, miR24‐2 inhibits JMJD2A by increasing miR6079 and then increases H3K9me3. Strikingly, miR24‐2 increases the expression of Pim1 dependent on H3K9me3 and METTL3. Notably, our findings suggest that miR24‐2 alters several related genes (pHistone H3, SUZ12, SUV39H1, Nanog, MEKK4, pTyr) and accelerates progression of liver cancer cells through Pim1 activation. In particular, Pim1 is required for the oncogenic action of miR24‐2 in liver cancer. This study elucidates a novel mechanism for miR24‐2 in liver cancer and suggests that miR24‐2 may be used as novel therapeutic targets of liver cancer.

## 1 INTRODUCTION

Primary liver cancer (PLC) is a common cancer with high morbidity and high mortality.^1^ The three most common subtypes of PLC include the following: hepatocellular carcinoma (HCC), cholangiocarcinoma (CC) and combined tumour (combined HCC/CC, CHC). Recent studies have shown that the occurrence and development of liver cancer are related to the self‐renewal of liver cancer stem cells.^2^

miRNAs are a class of conserved small non‐coding RNAs,^3^ and it ultimately becomes functional RNAs and regulates the expression of multiple genes through transcription, nuclear maturation and cytoplasmic processing.^4^ The mature miRNA binds to members of the argonaute protein family to form an RNA‐induced silencing complex (RISC), which in turn silences the expression of its complementary target messenger RNA.^5^ Moreover, abnormal miRNAs lead to the development of certain disease.^6^

Several microRNAs are associated with carcinogenesis and tumour progression. miR‐24‐2 is expressed in various tissues of human body and participates in various physiological processes such as erythropoiesis,^7^ lipogenesis,^8^ T‐cell senescence^9^ and osteoblast differentiation^10^ and can also participate in regulatory cellular growth, proliferation, apoptosis^11^, ^12^ and DNA damage repair pathways.^13^ In particular, miR‐24‐2 has a dual role of carcinogenicity and tumour suppression.^14^ Recently, miR‐23a/24‐2/27a cluster promotes hepatic metastasis.^15^ Moreover, miR24‐2 inhibits B‐cell and acute leukaemia cell.^16^, ^17^ Furthermore, a study provides new insights about the roles of the miR‐24‐2 that it helps to maintain muscle mass.^18^ miR‐24‐2 was overexpressed on the proteome of 70Z/3 pre‐B lymphoblast cells^19^ and muscle hypertrophic stimuli.^20^ In addition, preferential star strand biogenesis of miR‐24‐2 targets protein kinase C and suppresses cell survival in breast cancer cells.^14^ Moreover, miR24‐2 controls the expression and functions of several genes in cancer, for example NOTCH1, PIP4K2B, DIP2B and insulin‐like growth factor binding protein‐5 (IGFBP5).^12^, ^21^ Another research suggests the protein arginine methyltransferase 7 (PRMT7) repressed by miR‐24‐2 controls pluripotent factors Oct4, Nanog, Klf4 and c‐Myc.^22^, ^23^ Furthermore, miR24‐2 may act as tumour biomarker.^24^, ^25^, ^26^ Strikingly, the amplification of miR24‐2 was confirmed in gastric cancer (GC).^27^ Furthermore, miR24‐2 is associated with apoptosis in cancer cells. For examples, miR‐24‐2 may act as negative regulators of BCL2 in cancer cell.^28^, ^29^

In this study, we indicate that miR24‐2 and Pim1 are up‐regulated in human liver cancers, and miR24‐2 accelerates growth of liver cancer cells in vitro and in vivo. Moreover, oncogene Pim1 is required for the oncogenic action of miR24‐2 in human liver cancer. This study elucidates a novel mechanism for miR24‐2 in liver cancer cells.

## 2 MATERIALS AND METHODS

### 2.1 Liver cancer patients

Human primary liver cancer tissues used for analysis were obtained from liver cancer patients who had undergone surgery. Informed consent was obtained from all subjects. All patients were diagnosed as liver cancer according to histological examination by at least three pathologists or clinicians.

### 2.2 Cell lines and plasmids

Human hepatoma cell lines Hep3B were obtained from the Cell Bank of Chinese Academy of Sciences (Shanghai, China). These cell lines were maintained in Dulbecco's modified Eagle's medium (Gibco BRL Life Technologies) supplemented with 10% foetal bovine serum (Gibco BRL Life Technologies). Lentivirus rLV‐miR, rLV‐miR24‐2 and rLV‐miR6079 were purchased from Wu Han Viral Therapy Technologies Co. Ltd. pRFP‐C‐RS and pRFP‐C‐RS‐Pim1 were purchased from Origene.

### 2.3 Cell infection and transfection

Cells were infected with lentivirus and transfected with DNA plasmids according to the manufacturer's instructions and our pervious protocol.^30^, ^31^

### 2.4 RT‐PCR

Total RNA was purified using Trizol (Invitrogen) according to the manufacturer's instructions and our pervious protocol.^32^, ^33^ Gene primer: Pim1: P1:5′‐GAGTGGATCCGCTACCATCG‐3′; P2:5′‐TACTCGGGAAGCTGGAGACA‐3′. JMJD2A:P1:5′‐GGATAATGACCTTTTATCCA‐3′; P2:5′‐TCTCCAGCCTCTTGAGTCAC‐3′. Src: P1:5′GGAGACAGACCTGTCCTTCA‐3′; P2:5′‐GTAGGCCACCAGCTGCTGCA‐3′. β‐Actin was used as an internal control.

### 2.5 MicroRNA detection

Real‐time RT‐PCR‐based detection of mature miR24‐2, miR6079 and U6 snRNA was achieved with the miRNA Detection kit (including a universe primer, U6 primers) (Qiagen) and miR24‐2‐specific upstream primers (5′‐TGCCTACTGAGCTGAAACACAG‐3′) and miR6079‐specific upstream primers (5′‐TTGGAAGCTTGGACCAACTAGCTG‐3′).^33^

### 2.6 Western blotting

Samples were separated on a 10% sodium dodecyl sulphate‐polyacrylamide gel electrophoresis (SDS‐PAGE) and transferred onto nitrocellulose membranes and then blocked in 10% dry milk‐TBST (20 mmol/L Tris‐HCl [PH 7.6], 127 mmol/L NaCl, 0.1% Tween‐20) for two hours at 37°C. Following three washes in Tris‐HCl pH 7.5 with 0.1% Tween 20, the blots were incubated with primary antibody overnight at 4°C. Following three washes, membranes were then incubated with secondary antibody overnight at 4°C. Primary antibodies include the following: anti‐glypican‐3 (Abcam), anti‐PCNA (Abcam), anti‐METTL3 (Abcam), anti‐histone H3 (Abcam), anti‐H3K9me3 (Abcam), anti‐JMJD2A (Santa Cruz, Biotech), anti‐Pim1 (Santa Cruz, Biotech), pHistone H3 (Abcam), anti‐SUZ12 (Santa Cruz, Biotech), anti‐SUV39H1 (Santa Cruz, Biotech), anti‐Nanog (Abcam), anti‐MEKK4 (Abcam), anti‐pTyr (Abcam). Signals were visualized by enhanced chemiluminescence plus kit (GE Healthcare) according to our pervious protocol.^34^

### 2.7 Northern–Western blotting for miRNA

RNA samples were separated on 12% polyacrylamide/8M urea gel. Soak Hybond‐N+ membrane (Amersham Pharmacia) in ddH2O for a few seconds and in transfer buffer (0.5× TBE) for 20 minutes and soak two pieces of Whatman paper in 0.5× TBE. Separated RNA in gel was electro‐blotted onto Hybond‐N+ membrane (Amersham Pharmacia). After UV cross‐linking and air‐drying, blotted membrane was prehybridized with hybridization buffer at 42°C for 60 min and then hybridized with biotin‐labelled antisense miR24‐2 probe and incubated at 42°C for overnight. The membrane was washed 4 times at 42°C with 2× SSC and 0.5% SDS and then Western blotting with anti‐biotin according to our pervious protocol.^35^

### 2.8 Co‐immunoprecipitation (IP)

The protein is pre‐cleared with protein G/A‐plus agarose beads (Santa Cruz, Biotechnology, Inc) for 1 hour at 4°C and the supernatant is obtained after centrifugation (3000 *g*) at 4°C. The pre‐cleared supernatant is incubated with primary antibody or normal IgG by rotation for 4 hours at 4°C, and then, the immunoprecipitates are incubated with protein G/A‐plus agarose beads by rotation overnight at 4°C and then centrifuged at 3000 *g* for 5 min at 4°C. The precipitates are washed five times × 3 min with beads wash solution and then resuspended in sample loading buffer to incubate for 5 min at 100°C. Ultimately, Western blot was performed with the related antibody according to our pervious protocol.^36^

### 2.9 RNA Immunoprecipitation (RIP)

Ribonucleoprotein particle‐enriched lysates were incubated with protein A/G‐plus agarose beads (Santa Cruz, Biotechnology, Inc) together with the primary antibody or normal IgG for 4 hours at 4°C. Beads were subsequently washed five times with 50 mmol/L Tris‐HCl (pH 7.0), 150 mmol/L NaCl, 1 mmol/L MgCl_2_ and 0.05% NP‐40 and twice after addition of 1M urea. Finally, RT‐PCR was performed according to the manufacturer's instructions and our pervious protocol.^37^, ^38^

### 2.10 Dual‐luciferase reporter assay

Cells were transiently transfected with luciferase construct plasmids with the use of the Lipofectamine^TM^ 2000 (Invitrogen). After incubation for 36‐48 hours, the cells were harvested with Passive Lysis Buffer (Promega), and luciferase activities of cell extracts were measured with the use of the dual‐luciferase assay system (Promega) according to the manufacturer's instructions and our pervious protocol.^39^, ^40^

### 2.11 Cell proliferation CCK8 assay

CCK8 assay according to the manufacturer's instructions and our pervious protocol.^41^ In brief, cells at a concentration 2 × 10^4^ were seeded into 96‐well culture plates in 100 μL culture medium containing 10% heat‐inactivated foetal calf serum (FCS). Before detected, add 10 μg/well cell proliferation reagent CCK8 and incubate for 4 hours at 37°C in 5% CO_2_ humidified incubator.

### 2.12 BrdU staining

Cells were cultured for 48 hour before treatment with 10 μL BrdU (Roche) for 4 hours. Thereafter, the immunofluorescent staining with an anti‐BrdU antibody was performed according to the manufacturer's instructions (Becton Dickinson) and our pervious protocol.^42^

### 2.13 Colony formation efficiency assay

Cells were plated on a 10‐cm dish (Corning Inc), and the 10 mL DMEM containing 10% FBS was added into each dish of the three replicate. Then, these dishes were incubated at 37°C in 5% CO_2_ humidified incubator for ten days at least. Cell colonies on the dish were stained with Crystal Violet (Henan Tianfu Chemical Co., Ltd.), and the colonies were counted according to the manufacturer's instructions and our pervious protocol.^32^, ^43^

### 2.14 Cells sphere formation ability assay

Cells were collected and subsequently cultured in ultra low attachment 6‐well plates (Corning Inc) at a density of no more than 5000 cells/well. The sphere from ten random chosen fields of at least three independent samples was counted according to the manufacturer's instructions.

### 2.15 Xenograft transplantation in vivo

Four‐week male athymic Balb/C mice were purchased from Shi Laike Company. The athymic Balb/C mice were injected at the armpit area subcutaneously with suspension of transfected cancer cells. The mice were observed over 4 weeks at least and then killed to recover the xenograft. The use of mice for this work was reviewed and approved by the institutional animal care and use committee in accordance with China national institutes of health guidelines.^44^

## 3 RESULTS

### 3.1 The expression of miR24‐2 is up‐regulated in human liver cancer tissues

**Figure 1:**
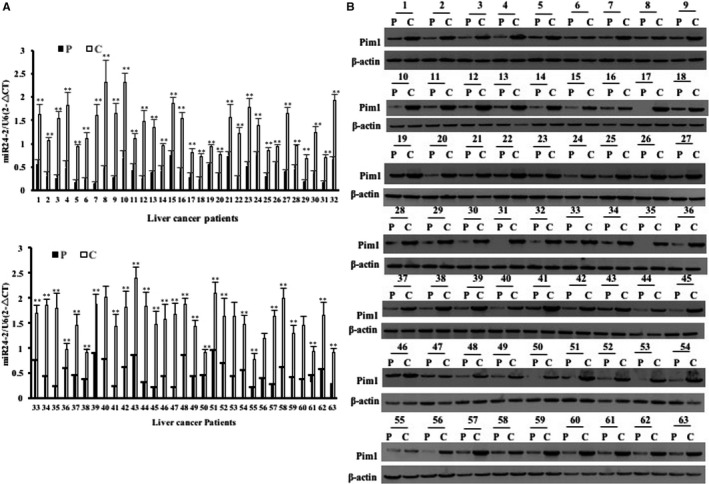
Expression analysis of miR24‐2 and Pim1 in human primary liver cancer tissue. A, The analytic results of real‐time RT‐PCR for mature miR24‐2 in liver cancer tissue (C) and its para‐cancerous liver tissues (P), respectively. B, The analytic results of Western blotting with anti‐Pim1 in human primary liver cancer tissue (C) and its para‐cancerous liver tissues (P), respectively. β‐Actin as internal control

To validate the relationship between miR24‐2 and Pim1, we detected the expression of miR24‐2 and Pim1 in the patients of liver cancer. Real‐time RT‐PCR showed that the mature miR24‐2 was significantly increased in the liver cancer tissues compared to their adjacent noncancerous tissues (100%, n = 63, *P* < .01) (Figure 1A). Northern‐Western blotting also showed that the mature miR24‐2 was significantly increased in the liver cancer tissues compared to their adjacent noncancerous tissues (100%, n = 63, *P* < .01) (Figure S1). The Pim1 mRNA was significantly increased in liver cancer tissues compared to their adjacent noncancerous tissues (Figure S2) [96.83% (61/63), n = 63, *P* < .01]. Western blotting with anti‐Pim1 showed that the expression of Pim1 was significantly increased in liver cancer tissues compared to their adjacent noncancerous tissues (Figure 1B) [96.83% (61/63), n = 63, *P* < .01]. Furthermore, the Src mRNA was significantly increased in liver cancer tissues compared to their adjacent noncancerous tissues (Figure S3A) (100%, n = 27, *P* < .01). Western blotting with anti‐Src showed that the expression of Src was significantly increased in liver cancer tissues compared to their adjacent noncancerous tissues (Figure S3B) (100%, n = 27, *P* < .01). Immunohistochemical staining showed that the Src expression was significantly increased in liver cancer tissues compared to their adjacent noncancerous tissues (100%, n = 60, *P* < .01) (Figure S3Ca,b). Moreover, there was a strong positive relevance between miR24‐2 and Pim1 or Src in human liver cancer. Collectively, these observations suggest both miR24‐2 and Pim1 or Src are up‐regulated in human liver cancers.

### 3.2 miR24‐2 accelerates growth of liver cancer cells in vitro and in vivo

**Figure 2:**
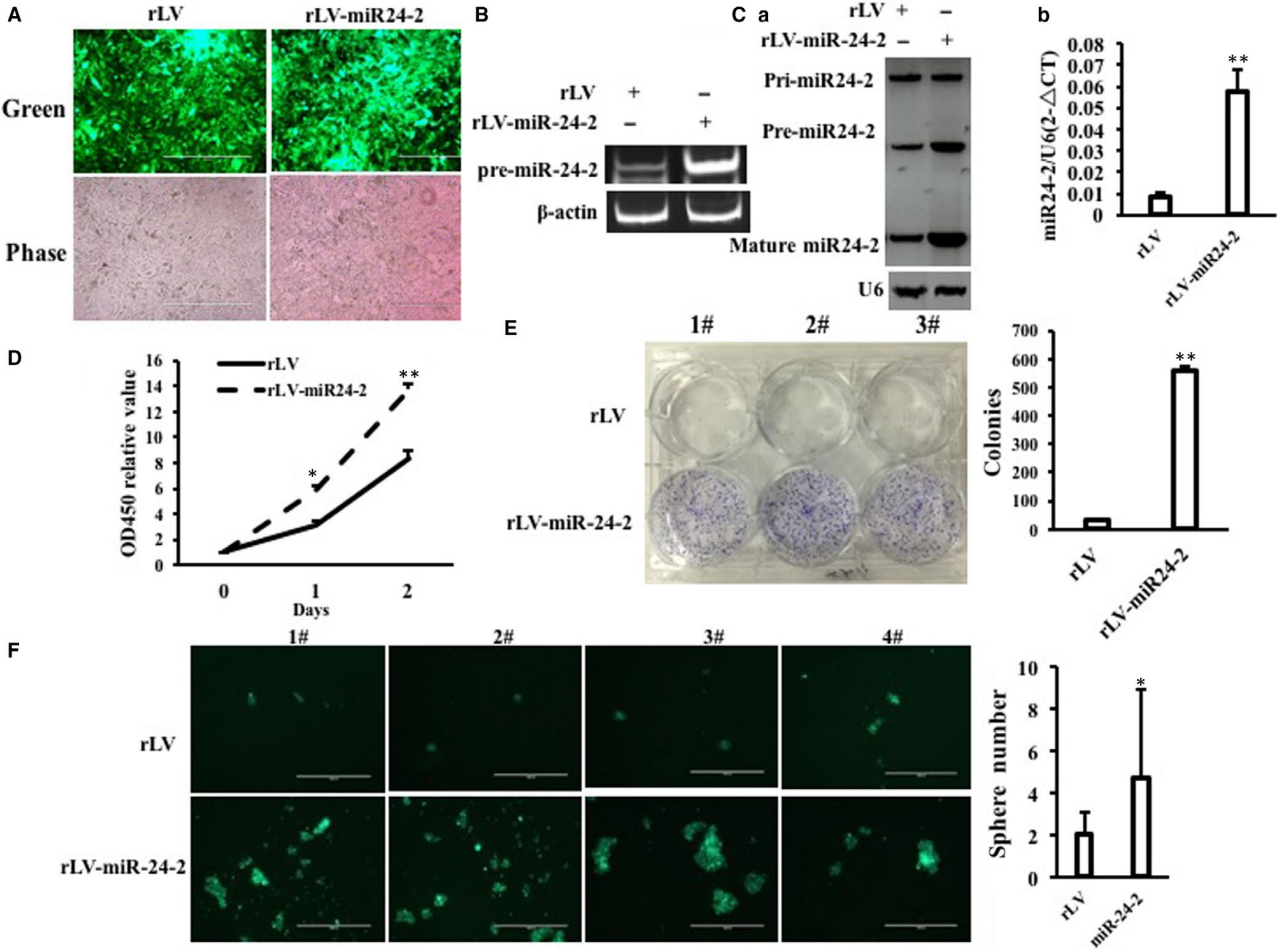
miR24‐2 accelerates growth of liver cancer cells in vitro. A, Green protein is observed under a fluorescence microscope in the two stable Hep3B cell lines by infecting with rLV or rLV–miR24‐2, respectively (original magnification ×100, scale bars, 400 μm). B, The RT‐PCR analysis for pre‐miR24‐2 in the two stable Hep3B cell lines by infecting with rLV or rLV–miR24‐2, respectively. β‐Actin as internal control. C, a, The Northern‐Western blot analysis for miR24‐2 in the two stable Hep3B cell lines by infecting with rLV or rLV–miR24‐2, respectively. U6 as internal control. b, The real‐time RT‐PCR analysis for mature miR24‐2 in the two stable Hep3B cell lines by infecting with rLV or rLV–miR24‐2, respectively. U6 as internal control. Each value was presented as mean ± standard error of the mean (SEM) (Student's *t* test). Bar ± SEM. **P < .01; *P < .05. D, Cell proliferation assay. E, Colony formation assay. F, Cell sphere formation assay. Each value was presented as mean ± standard error of the mean (SEM) (Student's *t* test). Bar ± SEM. **P < .01; *P < .05

**Figure 3:**
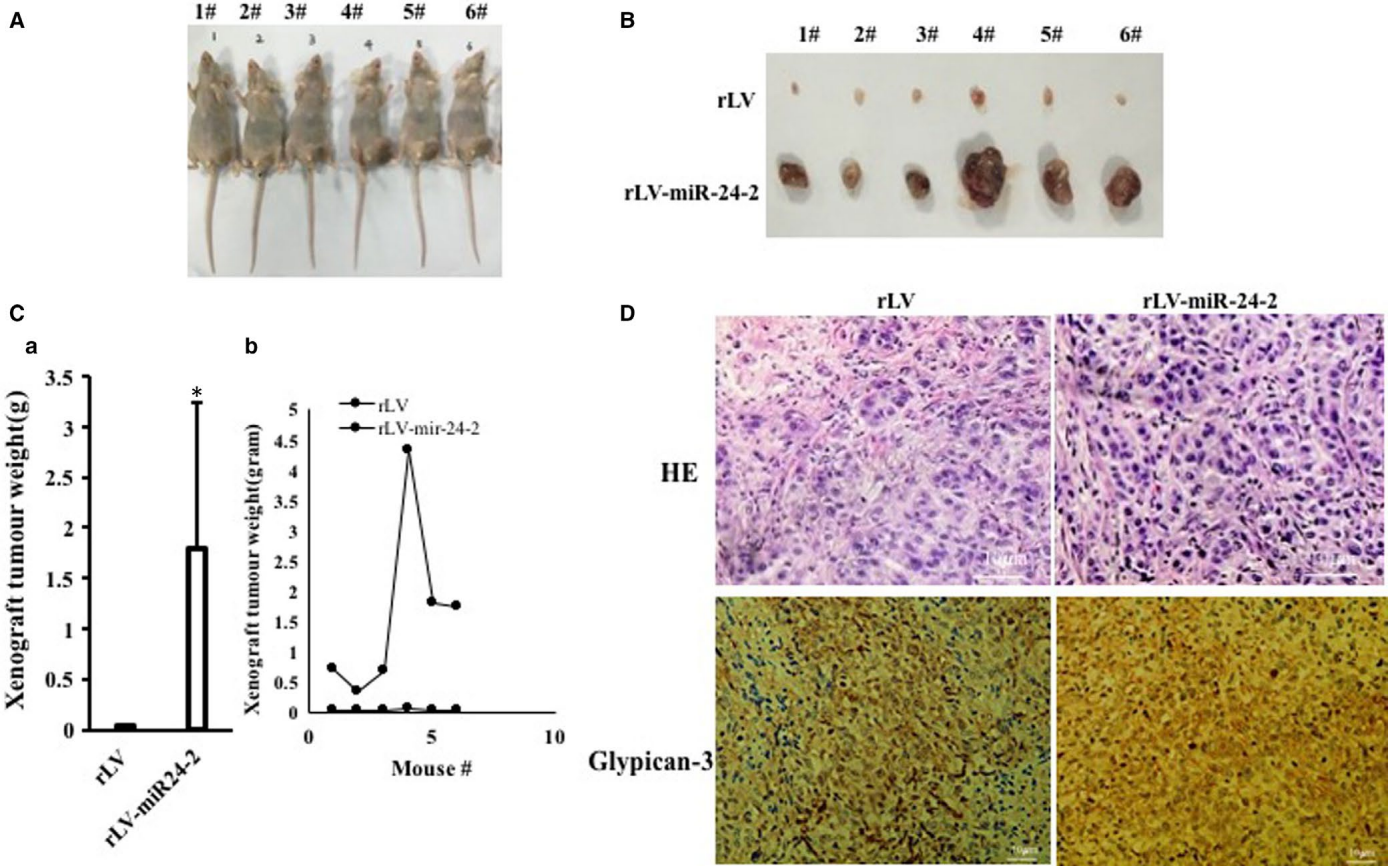
miR24‐2 accelerates growth of liver cancer cells in vivo*.* A, The photography of Balb/C nude mouse. B, The photography of xenograft tumours. C, The xenograft tumours weight (gram). Data were means of value from nine Balb/c mice, mean ± SEM, n = 6,*P < .05;**P < .01. D, Haematoxylin‐eosin (HE) staining and immunohistochemical staining for glypican‐3 in xenograft tumours (original magnification ×100)

To investigate whether miR24‐2 influences on growth of human liver cancer cells, we first constructed pLVX‐Zs‐Green‐miR24‐2 recombinant plasmid and prepared the rLV‐miR24‐2 lentivirus (Figure S4A,B). As shown in Figure 2A, the Green was found in two groups. The pre‐miR24‐2 was significantly overexpressed in rLV‐miR24‐2 group compared to rLV group (Figures 2B and S5), and mature miR24‐2 was significantly increased in rLV‐miR24‐2 group compared to rLV group (Figure 2Ca,b). Strikingly, we designed the outward‐facing primers for RT‐PCR for circ‐miR24‐2 (loop structure) and the results showed that circ‐miR24‐2 was increased in rLV‐miR24‐2 group compared to rLV group (Figure S6). Next, as shown in Figure 2D, the growth ability was significantly increased at the second days and the third days in rLV‐miR24‐2 group compared to rLV group, respectively (*P* < .01). The colony formation rate was significantly increased in rLV‐miR24‐2 compared to rLV group (5.66% ± 0.67% vs 92.99% ± 3.62%, *P* = .000389 < .01) (Figure 2E). Furthermore, the sphere formation rate was significantly increased in rLV‐miR24‐2 group compared to rLV group (2% ± 1.08% vs 4.69% ± 4.21%, *P* = .0259 < .05) (Figure 2F). To examine the effect of miR24‐2 on hepatocarcinogenesis in vivo, the stable liver cancer cell lines (rLV, rLV‐miR24‐2) were injected subcutaneously into Balb/C mice (Figure 3A). The mice were observed over 4 weeks and then killed to recover the xenografts (Figure 3B). As shown in (Figure 3C), the xenograft tumour weight was significantly increased in rLV‐miR24‐2 group compared to rLV group (0.028 ± 0.0206 grams vs 1.595 ± 1.459 grams, *P* = .0224 < .05). Moreover, xenograft tumours contain more of poorly differentiated cells in rLV‐miR24‐2 group compared to rLV group (Figure 3D). Collectively, these observations suggest that miR24‐2 promotes the growth ability of liver cancer.

### 3.3 miR24‐2 increases the mature miR6079 in liver cancer

**Figure 4:**
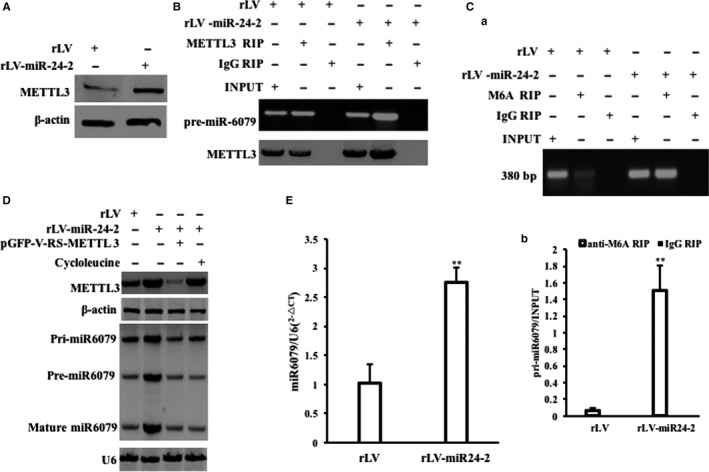
miR24‐2 increases the miR6079 expression via RNA methylation modification. A, Western blotting with anti‐METTL3 in Hep3B infected with rLV, rLV‐miR24‐2, respectively. B, RNA Immunoprecipitation (RIP) with anti‐METTL3 followed by RT‐PCR with pre‐miR6079 primers in Hep3B infected with rLV, rLV‐miR24‐2, respectively. Western blotting with anti‐METTL3 for each sample. C, a, RNA immunoprecipitation (RIP) with anti‐M6A followed by RT‐PCR with pre‐miR6079 primers. b, RNA immunoprecipitation (RIP) with anti‐M6A followed by real‐time RT‐PCR. D, Northern blotting analysis of miR6079 in Hep3B infected with rLV, rLV‐miR24‐2, rLV‐miR24‐2 plus pGFP‐V‐RS‐METTL3 and rLV‐miR24‐2 plus Cycloleucine, respectively. U6 as internal control. Western blotting with anti‐METTL3 for each sample and β‐actin as internal control. E, The real‐time RT‐PCR analysis. U6 as internal control. Each value was presented as mean ± standard error of the mean (SEM) (Student's *t* test). Bar ± SEM. **P < .01; *P < .05

Given that miR24‐2 accelerates the growth of human liver cancer cells, we consider to validate the effects of miR24‐2 on related signalling pathway in liver cancer. At the first time, the luciferase assay showed that the activity of N6‐adenosine‐methyltransferase 70‐kD subunit (METTL3) promoter luciferase reporter activity was significantly increased in rLV‐miR24‐2 group compared to rLV group (19 455.56 ± 5724.81 vs 212 848.13 ± 13 041.62, *P* = .00048 < .01) (Figure S7). However, the METTL3 3′UTR luciferase reporter activity was significantly not altered in rLV‐miR24‐2 group compared to rLV group (11 150.05 ± 3286.79 ± 3286.98 vs 10 361.71 ± 1293.24, *P* = .28525 > 0.05) (Figure S8). These results suggest that miR24‐2 promotes the transcription activity of METTL3 indirectly because METTL3 is not a direct target of miR24‐2. As shown in Figure 4A, the expression of METTL3 (a RNA methyltransferase) was significantly enhanced in rLV‐miR24‐2 group compared to rLV group. As shown in Figure 4B, the binding of METTL3 to Pre‐miR6079 was significantly enhanced in rLV‐miR24‐2 group compared to rLV group. Moreover, the level of methylation of pre‐miR6079 was significantly increased in rLV‐24‐2 group compared to rLV group (Figure 4Ca,b). In particular, the pri‐miR6079, pre‐miR6079 and mature miR6079 were significantly increased in rLV‐miR24‐2 group compared to rLV group. However, the pre‐mir6079 level was significantly not altered in rLV‐miR24‐2 plus pGFP‐V‐RS‐METTL3 and rLV‐miR24‐2 plus cycloleucine (a METTL3 inhibitor) compared to rLV control group (Figure 4D). Ultimately, mature miR6079 was significantly increased in rLV‐miR24‐2 group compared to rLV group (Figure 4E). Collectively, these observations suggest miR24‐2 enhances the expression of miR6079 via RNA methylation modification.

### 3.4 miR6079 inhibits JMJD2A and increases the tri‐methylation of histone H3 at Lys9

**Figure 5:**
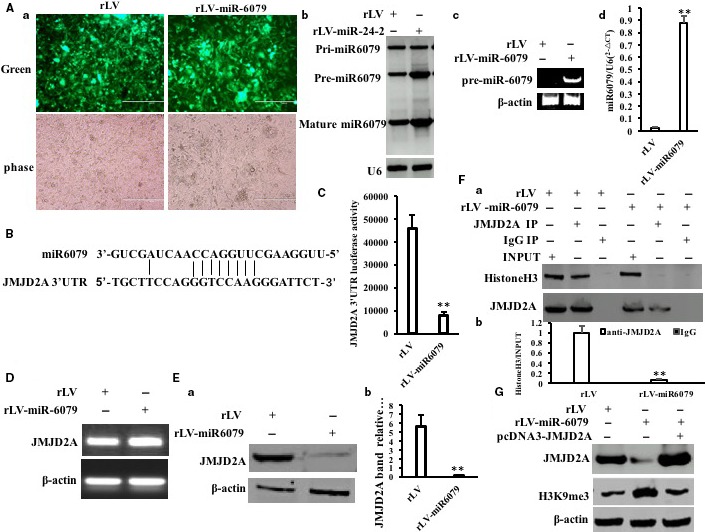
miR6079 enhances H3K9me3 by targeting for JMJD2A. A, a, the green protein is observed under a fluorescence microscope in the two stable Hep3B cell lines by infecting with rLV or rLV–miR6079, respectively (original magnification ×100, scale bars, 400 μm). B, The Northern‐Western blot analysis for miR‐6079 in the two stable Hep3B cell lines by infecting with rLV or rLV‐miR24‐2 respectively. U6 as internal control. c, The RT‐PCR analysis for pre‐miR6079. d, The real‐time RT‐PCR analysis for mature miR6079. Each value was presented as mean ± standard error of the mean (SEM) (Student's *t* test). Bar ± SEM. **P < .01; *P < .05. B, The informatics analysis of miR6079 targeting for JMJD2A 3′UTR using MirTarget scanning soft. C, The assay of JMJD2A 3′‐UTR luciferase activity. D, The RT‐PCR analysis for JMJD2A. β‐Actin as internal control. E, a, Western blotting with anti‐JMJD2A. b, Grey density analysis of band. F, a, Co‐immunoprecipitation (IP) with anti‐JMJD2A followed by Western blotting with anti‐histone H3. Western blotting with anti‐JMJD2A for each sample. b, Grey density analysis of band. G, Western blotting with anti‐H3K9me3 and anti‐JMJD2A. β‐Actin as internal control

To address whether miR6079 influences on the JMJD2A (a specific H3K9/36 me1/2/3 de‐methyltransferase), we established the two stable Hep3B cell lines by infecting with rLV or rLV–miR6079, respectively. As shown in Figure 5Aa, the Green was expressed in two groups. The pre‐miR6079 and mature miR6079 were significantly overexpressed in rLV‐miR6079 group compared to rLV group (Figure 5Ab). The pre‐miR6079 was significantly overexpressed in rLV‐miR6079 group compared to rLV group (Figure 5Ac) and mature miR6079 was significantly overexpressed in rLV‐miR6079 group compared to rLV group (Figure 5Ad). As shown in Figure 5B, mature miR6079 matches 3′ untranslational region (UTR) on JMJD2A mRNA via eight seed sequence. Moreover, the JMJD2A 3′UTR luciferase activity was significantly reduced in rLV‐miR6079 group compared to rLV control group (45 773.59 ± 6098.76 vs 8051.22 ± 1492.15, *P* = .00357 < .00414) (Figure 5C). Although the JMJD2A mRNA was not significantly altered between rLV‐miR6079 group and rLV control group (Figure 5D), the expression of JMJD2A was significantly decreased in rLV‐miR6079 group compared to rLV control group (Figure 5Ea,b). As shown in Figure 5F, the interplay between JMJD2A and histine H3 was significantly reduced in rLV‐miR6079 group compared to rLV control group. In particular, as shown in Figure S9, the interplay between JMJD2A and histone H3 was significantly reduced in rLV‐miR6079 group compared to rLV control group. However, the interplay between JMJD2A and histine H3 was significantly increased in miR6079 inhibitor group compared to rLV control group. Ultimately, the tri‐methylation of histone H3 at Lys9 (H3K9me3) was significantly increased in rLV‐miR6079 group compared to rLV group. However, the H3K9me3 was significantly reduced in rLV‐miR6079 plus pcDNA3‐JMJD2A (Figure 5G). Collectively, these findings suggest that miR6079 increases H3K9me3 by targeting for JMJD2A in liver cancer cells.

### 3.5 miR24‐2 enhances the tri‐methylation of histone H3 at Lysine (Lys) 9 dependent on miR6079

**Figure 6:**
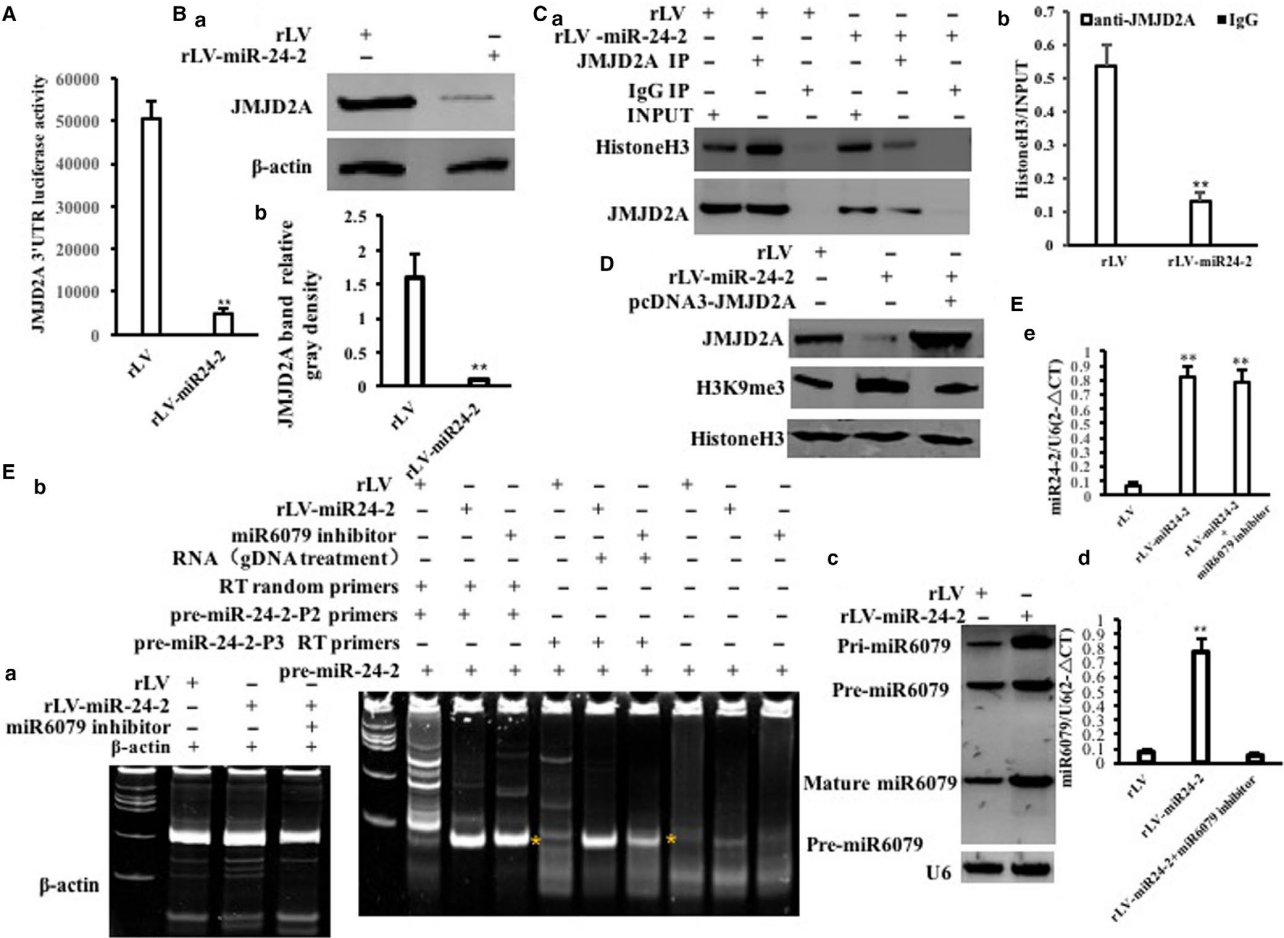
miR24‐2 enhances H3K9me3 by inhibiting JMJD2A A, The assay of JMJD2A 3′‐UTR luciferase activity. Each value was presented as mean ± standard error of the mean (SEM).**P < .01. B, a, Western blotting with anti‐JMJD2A. b, Grey density analysis of band. C, a, Co‐immunoprecipitation (IP) with anti‐JMJD2A followed by Western blotting with anti‐histone H3. Western blotting with anti‐JMJD2A for each sample. b, Grey density analysis of band. D. Western blotting with anti‐H3K9me3 and anti‐JMJD2A. β‐Actin as internal control. E, a. The RT‐PCR analysis for β‐actin. b. The RT‐PCR analysis for pre‐miR24‐2. c. The real‐time RT‐PCR analysis for mature miR24‐2. U6 as internal control. d. The real‐time RT‐PCR analysis for mature miR6079

**Figure 7:**
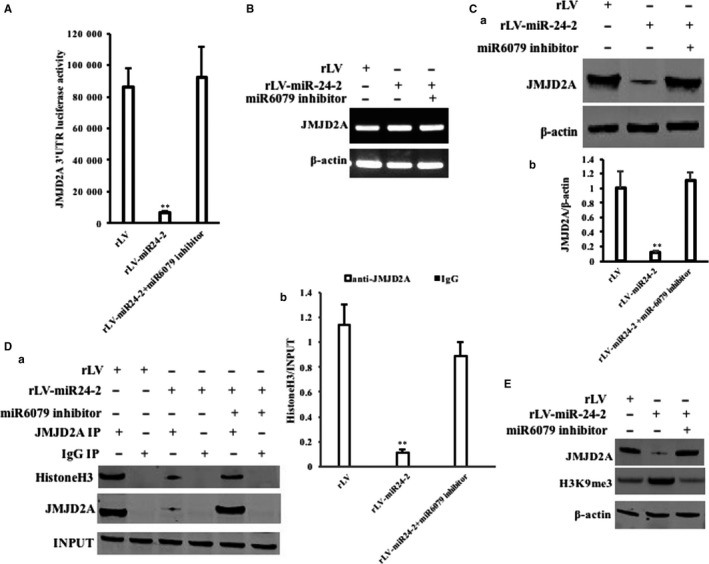
miR24‐2 enhances H3K9me3 by inhibiting JMJD2A dependent on miR6097. A, The assay of JMJD2A 3′‐UTR luciferase activity. Each value was presented as mean ± standard error of the mean (SEM).**P < .01. B, The RT‐PCR analysis for JMJD2A. C, a, Western blotting with anti‐JMJD2A. β‐Actin as internal control. b, Grey density analysis of bands. D, a, Co‐immunoprecipitation (IP) with anti‐JMJD2A followed by Western blotting with anti‐histone H3. b, Grey density analysis of band. E, Western blotting with anti‐H3K9me3 and anti‐JMJD2A. β‐Actin as internal control

Given that miR24‐2 promotes the expression of miR6079 which increases the H3K9me3 by targeting JMJD2A, we consider to explore whether miR24‐2 influences on tri‐methylation of histone H3 at Lys9 dependent on miR6079. As shown in Figure 6A, the JMJD2A 3′UTR luciferase activity was significantly reduced in rLV‐miR24‐2 group compared to rLV control group (50 668.87 ± 3771.17 vs 4793.75 ± 1134.86, *P* = .00096 < .01). The expression of JMJD2A was significantly decreased in rLV‐miR24‐2 group compared to rLV control group (Figure 6Ba,b). Furthermore, JMID2A was significantly reduced and mature miR24‐2 was overexpressed in rLV‐miR24‐2 group compared to rLV group (Figure S10A,B). As shown in Figure 6Ca,b, the interplay between JMJD2A and histine H3 was significantly reduced in rLV‐miR24‐2 group compared to rLV control. Ultimately, the H3K9me3 was significantly increased in rLV‐miR24‐2 group compared to rLV control group. However, the H3K9me3 was significantly reduced in rLV‐miR24‐2 plus pcDNA3‐JMJD2A (Figure 6D). As shown in Figure 6Ea,b, pre‐miR24‐2 was significantly increased in rLV‐miR24‐2 group and rLV‐miR24‐2 plus miR6079 inhibitor group. And mature miR24‐2 was significantly increased in rLV‐miR24‐2 group and rLV‐miR24‐2 plus miR6079 inhibitor group (Figure 6Ec). Mature miR6079 was significantly increased in rLV‐miR24‐2 group and decreased in rLV‐miR24‐2 plus miR6079 inhibitor group (Figure 6Ed). The JMJD2A 3′UTR luciferase activity was significantly reduced in rLV‐miR24‐2 group compared to rLV control group (85 997.3 ± 12 240.39 vs 6433.6 ± 1479.86, *P* = .003413 < .01). However, the JMJD2A 3′UTR luciferase activity was significantly not altered in rLV‐miR24‐2 plus miR6079 inhibitor group compared to rLV control group (85 997.3 ± 12 240.39 vs 92 438.56 ± 19 350.99, *P* = .3536 > 0.05) (Figure 7A). The JMJD2A mRNA was significantly not altered in rLV‐miR24‐2 group, rLV‐miR24‐2 plus miR6079 inhibitor group compared to rLV group (Figure 7B). Although the expression of JMJD2A was significantly decreased in rLV‐miR24‐2 group compared to rLV control group, it was increased in rLV‐miR24‐2 plus miR6079 inhibitor group compared to rLV control group (Figure 7Ca,b). JMJD2A was significantly reduced in rLV‐miR24‐2 group and was significantly not altered in rLV‐miR24‐2 plus miR6079 inhibitor group compared to rLV group, respectively (Figure S11A), and the mature miR24‐2 was increased in rLV‐miR24‐2 group compared to rLV group, respectively (Figure S11B). Moreover, the miR6079 was significantly increased in rLV‐miR24‐2 group and was not significantly altered in rLV‐miR24‐2 plus miR6079 inhibitor group compared to rLV group, respectively (Figure S10C). As shown in Figure7Da,b, although the interplay between JMJD2A and histine H3 was significantly reduced in rLV‐miR24‐2 group compared to rLV control, it was significantly not altered in rLV‐miR24‐2 plus miR6079 group compared to rLV control. Ultimately, the H3K9me3 was significantly increased in rLV‐miR24‐2 group compared to rLV group. However, the H3K9me3 was significantly not altered in rLV‐miR24‐2 plus miR6079 inhibitor group compared to rLV control group (Figure 7E). Collectively, these observations suggest that miR24‐2 enhances H3K9me3 by inhibiting JMJD2A dependent on miR‐6097.

### 3.6 miR24‐2 enhances Pim1 through H3K9me3

**Figure 8:**
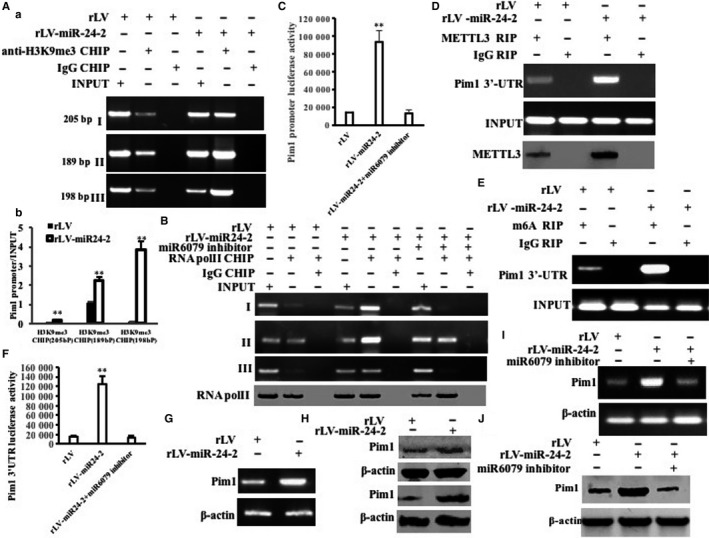
miR24‐2 enhances Pim1 through H3K9me3 and METTL3. A, a, Chromatin immunoprecipitation (CHIP) with anti‐H3K9me3 followed by PCR with Pim1 promoter primers. b. Real‐time CHIP analysis. Each value was presented as mean ± standard error of the mean (SEM).**P < .01. B, Chromatin immunoprecipitation (CHIP) with anti‐RNA pol II followed by PCR with Pim1 promoter primers. IgG CHIP as negative control. Pim1 promoter as INPUT. Western blotting with anti‐RNA pol II for each sample. C, The assay of Pim1 promoter luciferase activity. D, RNA immunoprecipitation (RIP) with anti‐METTL3 followed by RT‐PCR with Pim1 3′UTR primers. Western blotting with anti‐METTL3 for each sample. E, RNA immunoprecipitation (RIP) with anti‐M6A followed by RT‐PCR with Pim1 3′UTR primers. F, The assay of Pim1 3′UTR luciferase activity. G, The RT‐PCR for Pim1. β‐Actin as internal control. H, Western blotting with anti‐Pim1. β‐Actin as internal control. I, The RT‐PCR for Pim1. J, Western blotting with anti‐Pim1. β‐Actin as internal control

Given miR24‐2 enhances the modification of H3K9me3 and the expression of METTL3, we consider to address whether miR24‐2 enhances the Pim1 (a proto‐oncogene, serine/threonine kinase) by H3K9me3 and METTL3. As shown in Figure 8Aa,b, the H3K9me3 on the Pim1 promoter regions (region I, II, III) was significantly increased in rLV‐miR24‐2 group compared to rLV control group, respectively. However, miR6079 inhibitor abrogated this action of miR24‐2 (Figure S12). Moreover, as shown in Figure 8B, the RNA pol II on the Pim1 promoter regions (region I, II, III) was significantly increased in rLV‐miR24‐2 group compared to rLV group, respectively. However, miR6079 inhibitor abrogated this action of miR24‐2. As shown in Figure S13, super‐EMSA assay showed that the binding of RNA pol II to Pim1 promoter probe was significantly increased in rLV‐miR24‐2 group compared to rLV group; however, it was significantly not altered in rLV‐miR24‐2 plus miR6079 inhibitor group and was significantly decreased in rLV‐miR24‐2 inhibitor group compared to rLV group, respectively. And the luciferase activity of Pim1 promoter was significantly increased in rLV‐miR24‐2 group compared to rLV group (11 413.48 ± 1132.09 vs 94 103.17 ± 12 918.42, *P* = .0045961 < .01). However, the Pim1 promoter luciferase activity was significantly not altered in rLV‐miR24‐2 plus miR6079 inhibitor group compared to rLV group (11 413.48 ± 1132.09 vs 13 199.49 ± 3916.84, *P* = .3419 > 0.05) (Figure 8C). On the other hand, the interaction between METTL3 and Pim1 3′UTR (untranslational region) was significantly increased in rLV‐miR24‐2 group compared to rLV control group (Figure 8D). Moreover, the methylation on Pim1 32032UTR was significantly increased in rLV‐miR24‐2 group compared to rLV group (Figure 8E). However, miR6079 inhibitor abrogated this action of miR24‐2 (Figure S14). Furthermore, the luciferase activity of Pim1 3′UTR was significantly increased in rLV‐miR24‐2 group compared to rLV control group (24 492.27 ± 3669.02 vs 124 840.89 ± 16 835.57, *P* = .0049 < .01). However, the Pim1 3′UTR luciferase activity was not significantly altered in rLV‐miR24‐2 plus miR6079 inhibitor group compared to rLV group (24 492.27 ± 3669.02 vs 13 736.22 ± 2749.26, *P* = .3373 > 0.05) (Figure 8F). Therefore, the Pim1 mRNA was significantly increased in rLV‐miR24‐2 group compared to rLV group (Figure 8G). And the expression of Pim1 was significantly increased in rLV‐miR24‐2 group compared to rLV control group (Figure 8H). In particular, the Pim1 mRNA was significantly increased in rLV‐miR24‐2 group compared to rLV control group;, however, it was significantly not altered in rLV‐miR24‐2 plus miR6079 inhibitor group compared to rLV group (Figure 8I). Although the expression of Pim1 was significantly increased in rLV‐miR24‐2 group compared to rLV group, it was significantly not altered in rLV‐miR24‐2 plus miR6079 inhibitor group compared to rLV control group (Figure 8J). Collectively, these observations suggest that miR24‐2 increases the expression of Pim1 dependent on the modification of H3K9me3 in liver cancer cells.

### 3.7 Pim1 is required for the oncogenic action of miR24‐2

**Figure 9:**
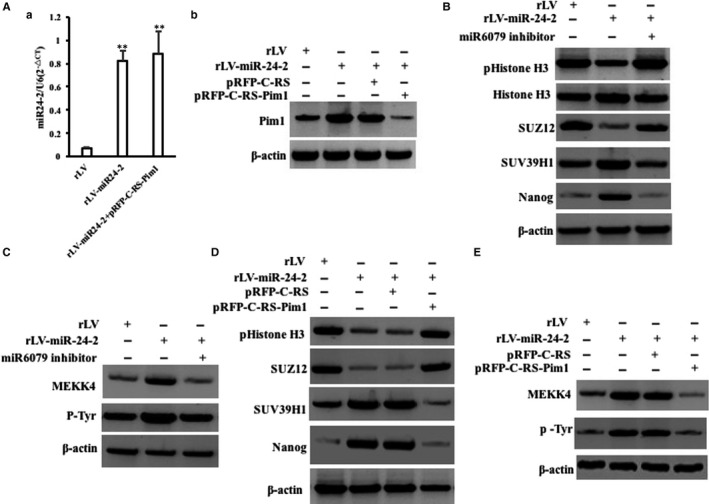
miR24‐2 alters the expression of related gene dependent on Pim1 A, a, The real‐time RT‐PCR analysis for mature miR24‐2. b, The Western blotting analysis with anti‐Pim1. β‐Actin as internal control. B, The Western blotting analysis with anti‐pHistone H3, anti‐histone H3, anti‐SUZ12, anti‐SUV39H1 and anti‐Nanog. β‐Actin as internal control. C, The Western blotting analysis with anti‐MEKK4, anti‐pTyr. D, The Western blotting analysis with anti‐Pim1, anti‐pHistone H3, anti‐SUZ12, anti‐SUV39H1 and anti‐Nanog. E, The Western blotting analysis with anti‐Pim1, anti‐MEKK4, anti‐pTyr. β‐Actin as internal control

Given that miR24‐2 enhances the expression of Pim1, we consider to identify whether Pim1 is required for the oncogenic action of miR24‐2 in liver cancer cells. Next, we performed the rescued test in rLV group, rLV‐miR24‐2 group and rLV‐miR24‐2 plus pRFP‐C‐RS‐Pim1 group. As sown in Figure 9Aa, miR24‐2 was significantly increased in rLV‐miR24‐2 group and rLV‐miR24‐2 plus pRFP‐C‐RS‐Pim1 group compared with rLV group, respectively. And the expression of Pim1 was significantly increased in rLV‐miR24‐2 group and decreased in rLV‐miR24‐2 plus pRFP‐C‐RS group compared with rLV group (Figure 9Ab). As shown in Figure 9B, the expression of pHistone H3 or SUZ12 was significantly decreased and the expression of SUV39H1 or Nanog was significantly increased in rLV‐miR24‐2 group compared to rLV control group, respectively. However, the expression of pHistone H3, SUZ12, SUV39H1 and Nanog was significantly not altered in rLV‐miR24‐2+miR6079 inhibitor group compared to rLV group, respectively. And the expression of MEKK4 or pTyr was significantly increased in rLV‐miR24‐2 group compared to rLV group, respectively. However, the expression of MEKK4 and pTyr was significantly not altered in rLV‐miR24‐2+miR6079 inhibitor group compared to rLV group, respectively (Figure 9C). Notably, although the expression of pHistone H3 or SUZ12 was significantly decreased and the expression of SUV39H1 or Nanog was significantly increased in rLV‐miR24‐2 group or rLV‐miR24‐2 plus pRFP‐C‐RS group compared to rLV group, respectively, it was significantly not altered in rLV‐miR24‐2 plus pRFP‐C‐RS‐Pim1 group compared to rLV group (Figure 9D). And the expression of MEKK4, pTyr was significantly increased in rLV‐miR24‐2 group and rLV‐miR24‐2 plus pRFP‐C‐RS compared to rLV group, respectively. However, the expression of MEKK4 and pTyr was significantly not altered in rLV‐miR24‐2 plus pRFP‐C‐RS‐Pim1 group compared to rLV group (Figure 9E).

**Figure 10:**
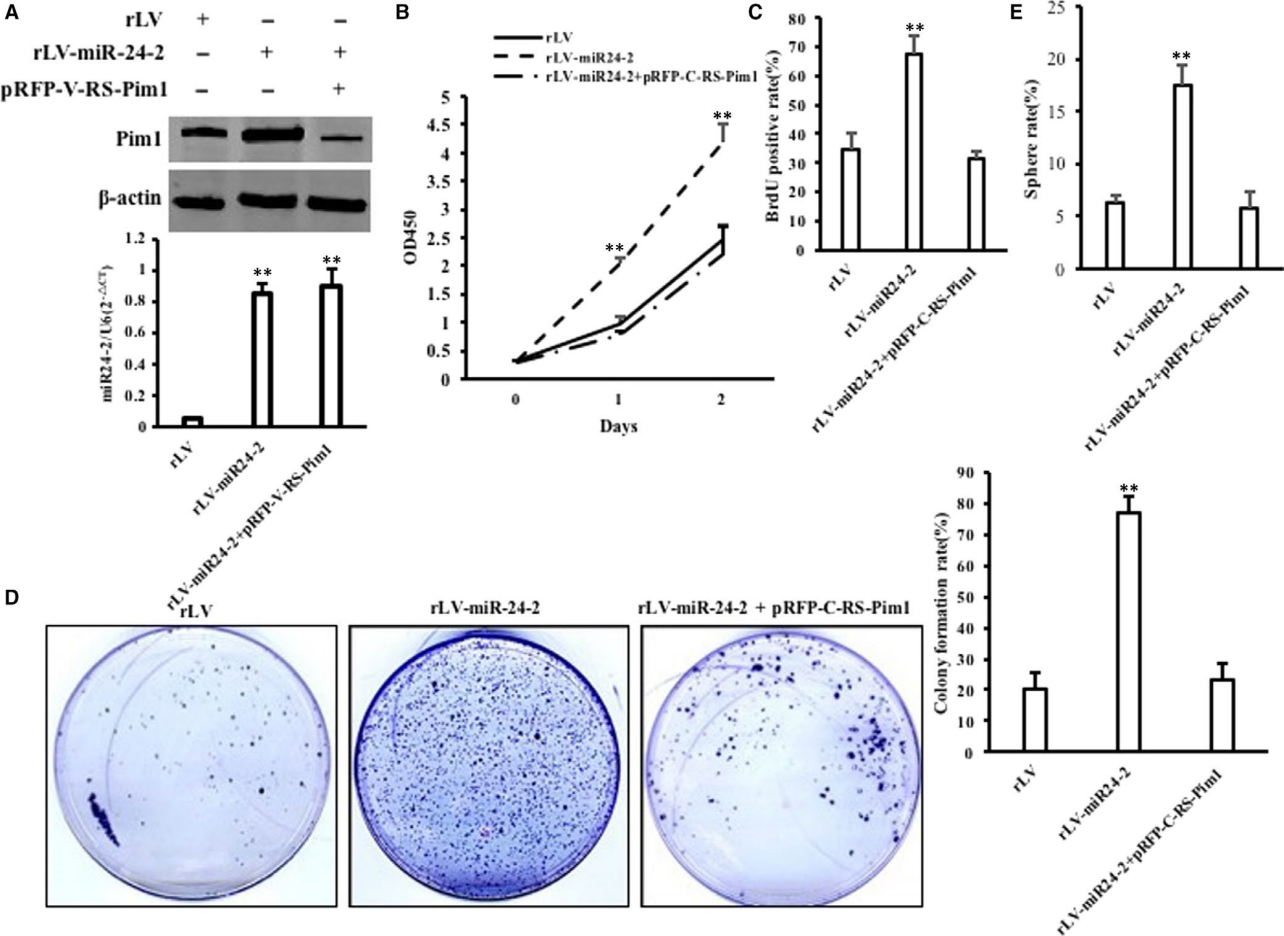
The rescued test for Pim1 in three stable Hep3B cell lines, including rLV group, rLV‐miR24‐2 group and rLV‐miR24‐2 plus pRFP‐C‐RS‐Pim1 group, respectively. A, a, The Western blotting analysis with anti‐Pim1. β‐Actin as internal control. B, The real‐time RT‐PCR analysis for mature miR24‐2. B, Cell growth assay. C, S phase cells assay using BrdU. D, Colony formation assay. a, Cell colony picture. b, Cell colony formation rate. Each value was presented as mean ± standard error of the mean (SEM) (Student's *t* test). Bar ± SEM. **P < .01; *P < .05. E, Cell sphere formation ability. Each value was presented as mean ± standard error of the mean (SEM) (Student's *t* test). Bar ± SEM. **P < .01; *P < .05

**Figure 11:**
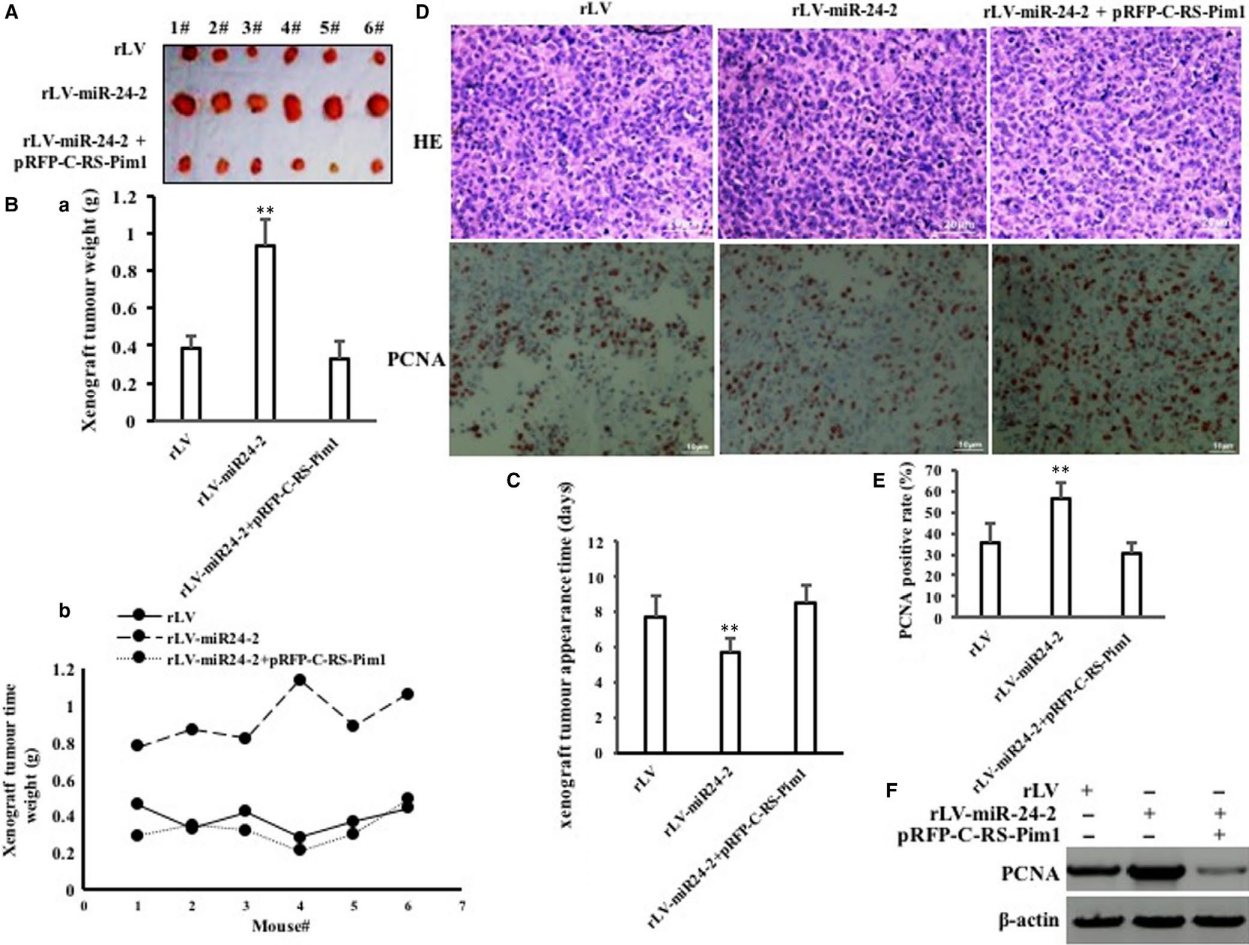
The rescued test for Pim1 in three stable Hep3B cell lines, including rLV group, rLV‐miR24‐2 group and rLV‐miR24‐2 plus pRFP‐C‐RS‐Pim1 group, respectively. A, The pictures of xenograft tumours from Balb/C nude mouse. B, (a‐b) The weight of xenograft tumours from Balb/C nude mouse. C, The appearance time of the xenograft tumours. Each value was presented as mean ± standard error of the mean (SEM) (Student's *t* test). Bar ± SEM. **P < .01; *P < .05. D, Haematoxylin‐eosin (HE) staining and anti‐PCNA staining of xenograft tumours (original magnification ×100). PCNA. E, The analysis of positive rate (%) of PCNA. F, The Western blotting analysis with anti‐PCNA. β‐Actin as internal control

Furthermore, the expression of Pim1 was increased in rLV‐miR24‐2 group and decreased in rLV‐miR24‐2 plus pRFP‐C‐RS‐Pim1 group compared to rLV group, and mature miR24‐2 was increased in rLV‐miR24‐2 group and rLV‐miR24‐2 plus pRFP‐C‐RS‐Pim1 group compared to rLV group (Figure 10A). As shown in Figure 10B, the growth of Hep3B was more rapid in rLV‐miR24‐2 group than in rLV group (*P* < .01). However, the growth of Hep3B was not significantly altered in rLV‐miR24‐2 plus pRFP‐C‐RS‐Pim1 group compared to Rlv group (*P* > .05). As shown in Figure 10C, the BrdU‐positive rate was significantly increased in rLV‐miR24‐2 group compared to rLV group (34.42% ± 5.57% vs 67.34% ± 6.24%, *P* = .0078 < .01). However, the BrdU‐positive rate of Hep3B was not significantly altered in rLV‐miR24‐2 plus pRFP‐C‐RS‐Pim1 group compared to rLV group (34.42% ± 5.57% vs 31.29% ± 2.46%, *P* = .1127 > 0.05). As shown in Figure 10D, although the colony formation ability was significantly increased in rLV‐miR24‐2 group compared to rLV group (20.48% ± 4.23% vs 77.34% ± 10.61%, *P* = .00825 < .01), it was significantly not altered in rLV‐miR24‐2 plus pRFP‐C‐RS‐Pim1 group compared to rLV group (20.48% ± 4.23% vs 23.3% ± 6.08%, *P* = .3576 > 0.05). As shown in Figure 10E, although the sphere formation rate was significantly increased in rLV‐miR24‐2 group compared to rLV group (6.25% ± 0.79% vs 17.48% ± 1.95%, *P* = .008991 < .01), it was significantly not altered in rLV‐miR24‐2 plus pRFP‐C‐RS‐Pim1 group compared to rLV group (6.25% ± 0.79% vs 5.69% ± 1.57%, *P* = .24676 > 0.05). As shown in Figures 11A,B and S15, the xenograft tumour weight was significantly increased in rLV‐miR24‐2 compared to rLV group (0.382 ± 0.069 g vs 0.927 ± 0.142 g, *P* = .0004375 < .01). However, the average xenograft tumour weight was significantly not altered in rLV‐miR24‐2 plus pRFP‐C‐RS‐Pim1 group compared to rLV group (0.382 ± 0.069 g vs 0.327 ± 0.093 g, *P* = .07246 > 0.05). Although the xenograft tumour appearance time was significantly decreased in rLV‐miR24‐2 compared to rLV group (7.67 ± 1.21 days vs 5.66 ± 0.816 days, *P* = .00898 < .01), it was significantly not altered in rLV‐miR24‐2 plus pRFP‐C‐RS‐Pim1 group compared to rLV group (7.67 ± 1.21 days vs 8.5 ± 1.05 days, *P* = .1446 > 0.05) (Figures 11C and S16). As shown in Figure 11D, PCNA‐positive rate was significantly increased in rLV‐miR‐24‐2 group compared to rLV group (35.54% ± 9.37% vs 56.19% ± 7.81%, *P* = .0085 < .01). However, the percentage of PCNA‐positive cells was significantly not altered in rLV‐miR24‐2 plus pRFP‐C‐RS‐Pim1 group compared to rLV group (35.54% ± 9.37% vs 30.46% ± 5.24%, *P* = .1349 > 0.05) (Figures 11D–F and S17). Collectively, these findings suggest that Pim1 determines the oncogenic functions of miR24‐2 in human liver cancer.

## 4 DISCUSSION

**Figure 12:**
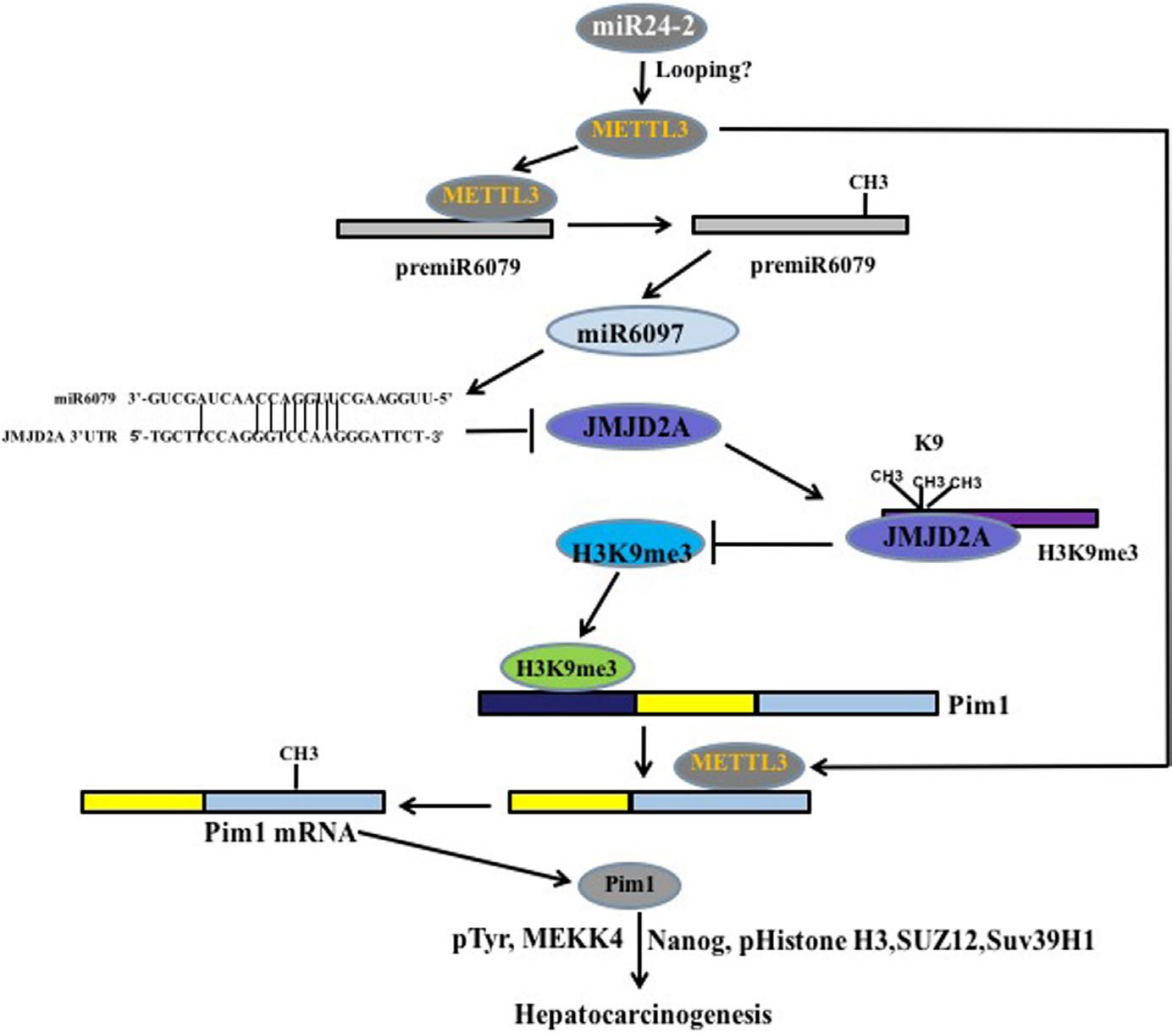
The schematic illustrates a model that miR24‐2 promotes progression of liver cancer cells by activating Pim1 through tri‐methylation of Histone H3 on the ninth lysine

Until now, studies have reported that miR‐24‐2 is involved in the development of several tumours; for examples, miRNA‐24‐2 accelerates the development of tumours such as gastric cancer and breast cancer by enhancing the expression of oncogene c‐Myc.^15^ And miR‐24‐2 has gradually become an important marker for predicting cancer prognosis and tumour progression.^45^, ^46^ Furthermore, miR‐24‐2 may act as an oncogene in clusters^47^ and promotes the metastasis of breast cancer.^15^ However, there are reports that miR‐24‐2 negatively regulates tumour cell growth under certain tumour microenvironments.^29^ In this study, as shown in Figure 12, our observations clearly indicate that miR‐24‐2 is highly expressed in human hepatocarcinoma tissues, which is related to the clinical features of patients. And miR‐24‐2 significantly promotes the growth ability of human hepatoma cells in vitro and in vivo. Moreover, the main mechanisms were found as follows: (a) miR‐24‐2 enhanced the expression of mRNA N6‐adenine methyltransferase METTL3 and increased the pri‐miR‐6079 methylation modification and its maturation depending on METTL3. (b) miR‐6079 increased H3K9me3 modification by targeting JMJD2A, thereby promoting H3K9me3 loading on the Pim1 promoter region and then increasing Pim1 transcriptional activity. (c) Pim1 determines the cancerous functions of miR‐24‐2. These results confirm that excess miR24‐2 can aggravate the malignant proliferation of human hepatoma cells.

Notably, our findings in this study provide a novel evidence for a novel active form of miR24‐2 in liver cancer. Our results showed that pre‐miR24‐2 may produce a loop structure. Besides the mature miR24‐2, pre‐miR24‐2 looping may play a key role in regulating and controlling the expression of several oncogenes. This mechanism is consistent with some reports.^48^ For examples, a recent study revealed that circRNA (circFOXP1) promoted proliferation of mesenchymal stem cells (MSCs)^49^ and circRNA (circFoxo3) regulated the RNA Pol II transcriptional complex in the nucleus.^50^ Thus, the mechanism how pre‐miR‐24‐2 is cyclized and plays roles remains to be further studied.

It is worth noting that this study demonstrates that overexpression of miR‐24‐2 in human hepatoma cell line Hep3B promotes the expression of N6‐adenine methyltransferase METTL3 and thereby increases methylation modification (m6A) on N6‐adenosine from specific RNA. Indeed, m6A is one of the major modifications of RNA that regulates the processing, translation and decay of RNA, which plays a key role in RNA metabolism and function.^51^, ^52^ Furthermore, m6A, which is mainly catalysed by METTL3, regulates RNA stability mainly through m6A‐specific binding proteins which in turn determines cellular fate and function.^53^, ^54^ In particular, N^6^‐methyladenosine (m^6^A) has been shown recently to play essential roles in various bioprocesses.^55^, ^56^, ^57^ Moreover, m6A RNA methylation regulates tumorigenesis^58^, ^59^ and controls stem cell self‐renewal and promotes cancer progression.^60^, ^61^ It indicates that the regulation of RNA modification by miR‐24‐2 plays an important role in the development of liver cancer.

Non‐coding RNA is also one of the important ways of epigenetic regulation. Significantly, our findings indicate that miR‐24‐2 can play a carcinogenic role together with miR‐6079 in human liver cancer cells. But, miR‐6079 is recently discovered and its functions are currently unclear in tumorigenesis. This study demonstrates that miR‐24‐2 can promote the transcriptional activity and maturation of pri‐miR‐6079 by increasing the m6A modification of pri‐miR‐6079 in human hepatoma cell Hep3B. This assertion is based on several observations: (a) the expression of N6‐adenosine‐methyltransferase METTL3 was significantly enhanced in rLV‐miR24‐2 group compared to rLV group. (b) miR24‐2 increases the interplay between METTL3 and pri‐miR6079. (c) miR24‐2 increases the level of RNA methylation of pri‐miR6079 dependent on METTL3. Moreover, our results suggest that miR6079 enhances H3K9me3 by targeting for JMJD2A. This evidence is based on results from three parallel sets of experiments: (a) Informatics analysis suggest that miR6079 may bind to seed sequence of JMJD2A 3′‐UTR. (b) miR6079 targets for JMJD2A and inhibits the expression of JMJD2A. (c) miR6079 increases the tri‐methylation of histone H3 on the ninth lysine (H3K9me3) dependent on JMJD2A. Therefore, the excessive miR‐24‐2 can inhibit the expression of JMJD2A via miR‐6079, thereby exerting its cancer‐promoting function. These results indicate that miR‐24‐2 can cooperate with miR‐6079 to promote the development and progress of liver cancer. Furthermore, bioinformatics prediction reveals that miR‐6079 also targets the 3′‐UTR region of some tumour‐associated gene mRNAs, such as glycine acetyltransferase GLYAT, CDKN2A binding protein CDKN2AIP, etc Therefore, miR‐24‐2 may also promote the development of liver cancer by affecting the expression of these genes. However, it should be studied further.

Obviously, our findings indicate that miR‐24‐2 inhibits the expression of histone demethylase JMJD2A and increased the H3K9me3 by promoting the maturation of miR‐6079 in human liver cancer cells. This evidence is based on results from three parallel sets of experiments: (a) JMJD2A 3′UTR luciferase activity was significantly reduced in miR24‐2 overexpressing Hep3B cell. (b) The expression of JMJD2 was significantly decreased in rLV‐miR24‐2 group compared to Rlv group. (c) H3K9me3 was significantly increased in rLV‐miR24‐2 group compared to rLV group. However, this action was abrogated by inhibiting miR6079 or increasing JMJD2A in miR24‐2 overexpressing Hep3B cells. It is well known that JMJD2A can catalyse the demethylation of histone ninth/36th lysine (H3K9/K36) and regulate the expression of certain genes at the epigenetic level.^62^ Studies have shown that JMJD2A is highly expressed in a variety of tumours^63^, ^64^, ^65^ and is involved in the regulation of cancer cell proliferation, survival and conversion between glycolysis metabolism and mitochondrial oxidation.^66^ However, several researches show that histone H3 demethylase JMJD2A drives tumorigenesis^65^, ^67^, ^68^, ^69^, ^70^, ^71^ and H3k9me3 was reduced in several cancers.^72^, ^73^ In particular, regulating H3K9me3 activity contributes to cancer progression.^74^ In fact, our present findings indicate miR‐24‐2 accelerates hepatocarcinogenesis by inhibiting JMJD2A and increasing H3K9me3.

Importantly, miR‐24‐2‐dependent miR‐6079 increased the level of tri‐methylation of histone lysine (H3K9me3) by inhibiting of JMJD2A. Studies have reported that H3K9me3 modification in the promoter region of the gene can affect the transcriptional activity of some genes.^75^, ^76^, ^77^ Our results also confirmed that miR‐24‐2 increased the H3K9me3 loading on the oncogene pim 1 promoter region, thereby promoting the expression of Pim1, indicating that the change of H3K9me3 modification triggered by miR‐24‐2 is beneficial to the growth of cancer cells at least in hepatoma cell Hep3B.

It has been confirmed that protein kinases can participate in most signal transduction in eukaryotic cells and control many cellular processes, including metabolism, transcription, cell cycle progression, apoptosis and differentiation, by adding phosphate groups to the substrate (phosphorylation) to alter the activity of the substrate, cellular localization or binding to other proteins.^78^, ^79^, ^80^ Of significance, in this study, miR24‐2 enhances Pim1 through H3K9me3 and METTL3. This evidence is based on results from six parallel sets of experiments: (a) miR‐24‐2 is associated with abnormal expression of Pim1 in human hepatoma cells. (b) miR24‐2 enhances the H3K9me3 loading on Pim1 promoter regions. (c) miR24‐2 enhances the transcriptional activity of Pim1. (d) miR24‐2 enhances the interplay between METTL3 and Pim1 3′UTR and increases the m6A methylation modification on the Pim13′UTR regions. Moreover, miR‐24‐2‐dependent miR‐6079 increased methylation of the Pim1 3′‐UTR region and promoted the regulatory activity of Pim1 3′‐UTR. (e) miR24‐2 enhances Pim1 3′UTR activity. (f) Although miR24‐2 increases the expression Pim1, it was abrogated by inhibiting miR6079 in miR24‐2 overexpressing Hep3B cells. Thus, miR‐24‐2 relies on miR‐6079 to facilitate transcription and translation of Pim1. Studies indicate that the serine/threonine protein kinase pim 1 is known to be an oncogene and is present in the cytoplasm and nucleus and is capable of phosphorylating different targets, most of which are involved in the cell cycle and apoptosis processes.^81^ Pim1 is overexpressed in various tumours such as breast cancer and prostate cancer and promotes tumour progression by promoting cell proliferation, survival and inhibition of apoptosis.^82^, ^83^, ^84^ Moreover, Pim1 promotes cancer cells growth by enhancing the Warburg effect^85^, ^86^ and promotes epithelial‐mesenchymal transition and oncogenesis.^87^, ^88^, ^89^, ^90^, ^91^ Therefore, miR‐24‐2 is likely to play a carcinogenic role by promoting the expression and function of Pim1 in liver cancer. Subsequently, it should further be proved in human liver cancer.

It is worth noting that our findings demonstrate pim 1 is required for the oncogenic action of miR24‐2 in liver cancer. This evidence is based on results from three parallel sets of experiments: (a) Although the expression of pHistone H3 or SUZ12 was significantly decreased and the expression of SUV39H1 or Nanog was significantly increased in rLV‐miR24‐2 group compared to rLV group, it was abrogated by inhibiting Pim1 in miR24‐2 overexpressing Hep3B cells. (b) Although the expression of MEKK4 and pTyr was significantly increased in rLV‐miR24‐2 group compared to rLV group, it was abrogated by inhibiting Pim1 in miR24‐2 overexpressing Hep3B cells. (c) miR24‐2 accelerates progression of liver cancer cells in vitro and in vivo. However, these functions were fully abrogated by inhibiting Pim1 in miR24‐2 overexpressing Hep3B cells. These related genes involved in this experiment had been reports. For examples, polycomb repressive complex 2 induces gene silencing through H3K27me3 in several cancers,^92^, ^93^, ^94^ and lysine methyltransferases SUV39H1 and Nanog regulate several oncogene expression in various cancer cells.^95^, ^96^ Recent work has provided a fact that the activation, regulation, and functions of MEKK kinases and tyrosine phosphorylation in several cancers.^97^, ^98^ Therefore, miR24‐2 may play an important role through these related genes during hepatocarcinogenesis.

In conclusions, this study revealed the novel mechanisms by which miR‐24‐2 plays a carcinogenic role in human hepatoma cells, but the detailed mechanism of specific processes remains to be further studied, including (a) in‐depth study of miR24‐2 should be performed in liver cancer, including the various stages of the maturation process and its direct targets in liver cancer. (b) The specific mechanism of miR‐24‐2 affecting epigenetic regulation needs further to be studied. (c) The related changes in histone modification and nucleic acid modification still need to be improved in liver cancer. (d) Further research is required in clinical applications, indicating whether miR‐24‐2 can be used as a clinical diagnostic indicator and targeting of therapeutic drugs for liver cancer patients. In a word, blocking miR24‐2 might represent a promising treatment strategy for human liver cancer.

## CONFLICTS OF INTEREST

The authors disclose no conflicts.

## AUTHORS' CONTRIBUTIONS

Dongdong Lu conceived the study and participated in the study design, performance, coordination and manuscript writing. Yuxin Yang, Shuting Song, QiuyuMeng, Liyan Wang, Xiaonan Li, Sijie Xie, Yingjie Chen, Xiaoxue Jiang, Chen Wang, Yanan Lu, Xiaoru Xin, Hu Pu, Xin Gui, Tianming Li, Jie Xu, Jiao Li, Song Jia performed the research. All authors have read and approved the final manuscript.
