# Supplementary material for: miR24‐2 accelerates progression of liver cancer cells by activating Pim1 through tri‐methylation of Histone H3 on the ninth lysine
Source: J Cell Mol Med. 2020 Feb 6;24(5):2772–90. doi: 10.1111/jcmm.15030 (PMC7077597; doi:10.1111/jcmm.15030)
Supplement: Supplementary file 1 [file JCMM-24-2772-s001.docx]

**
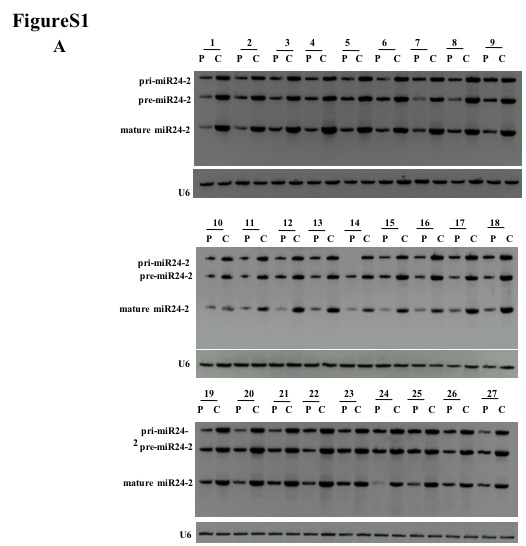
**

**
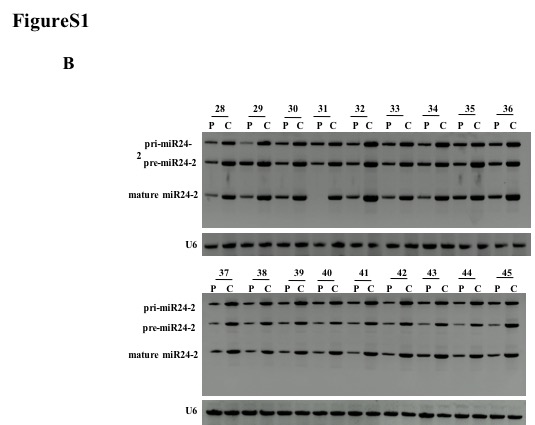
**

**
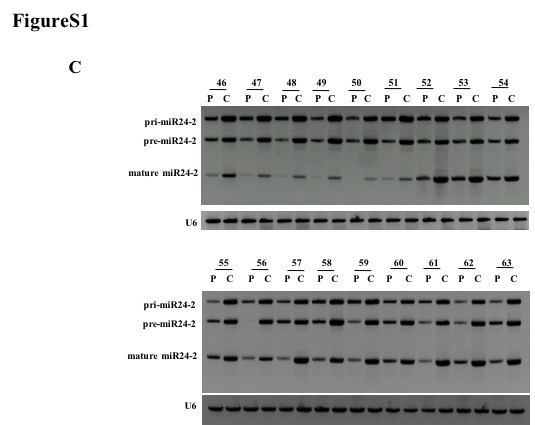
**

**
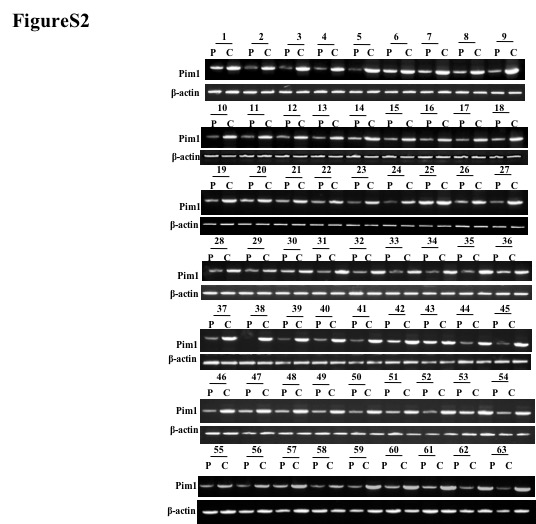
**

**
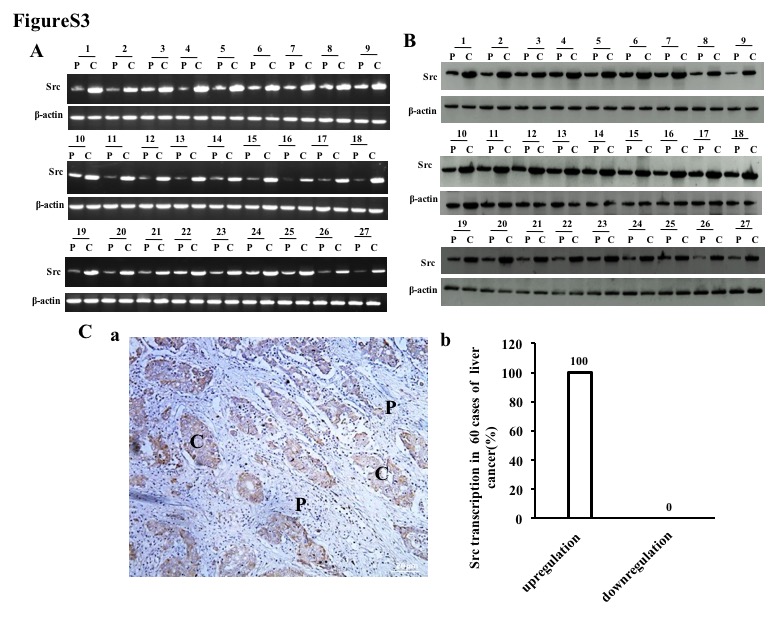

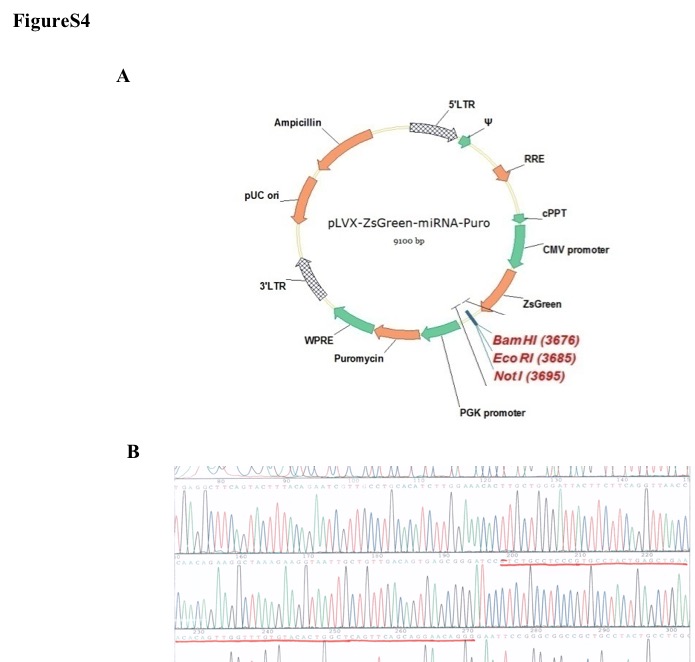
**

**
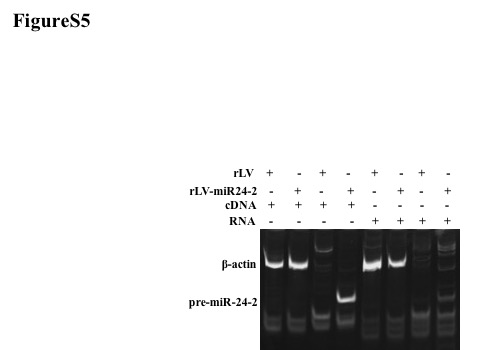
**

**
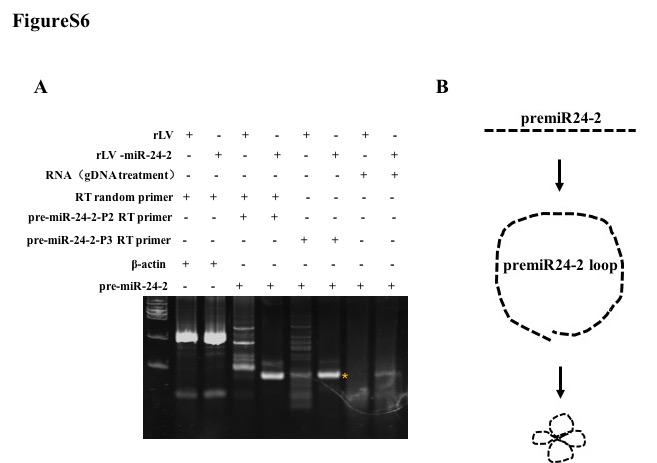
**

**
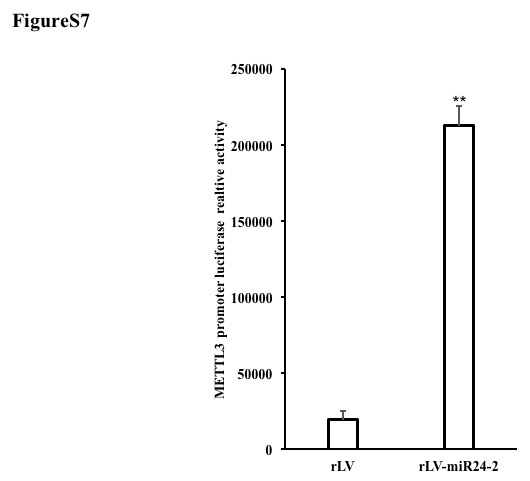
**

**
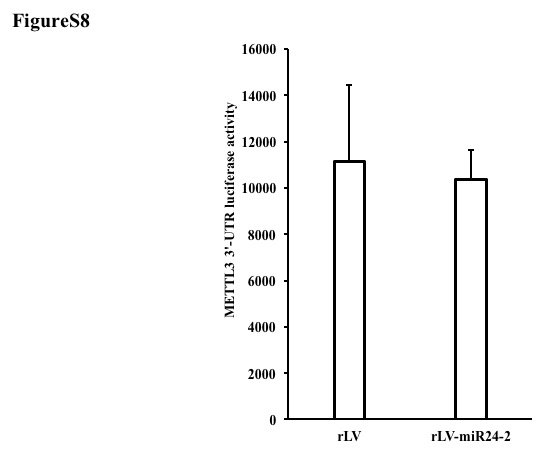
**

**
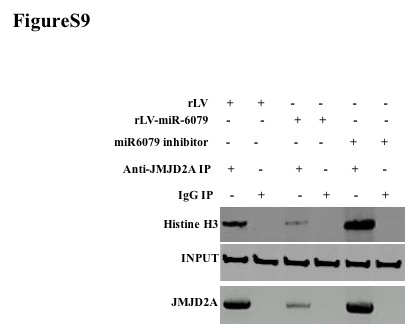
**

**
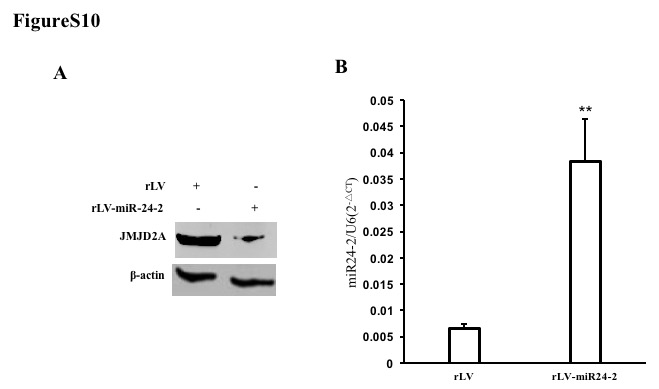
**

**
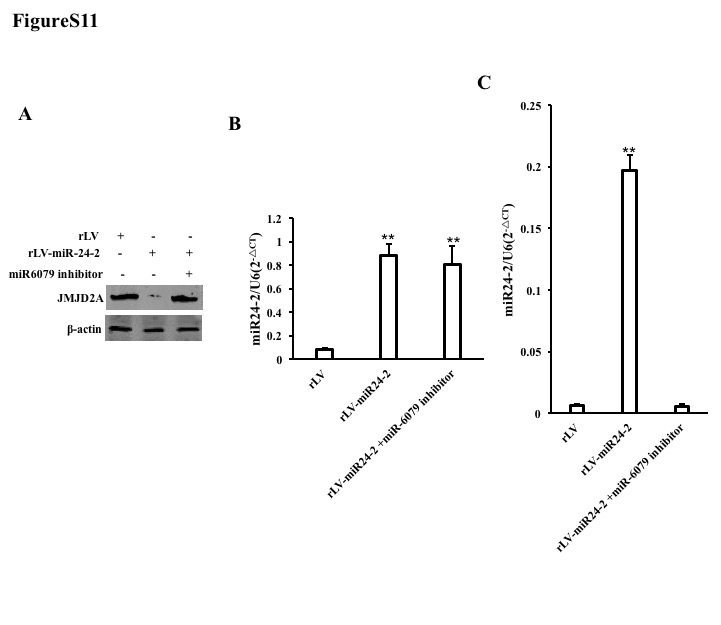
**

**
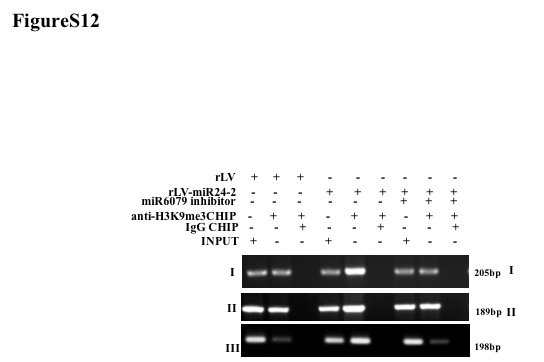
**

**
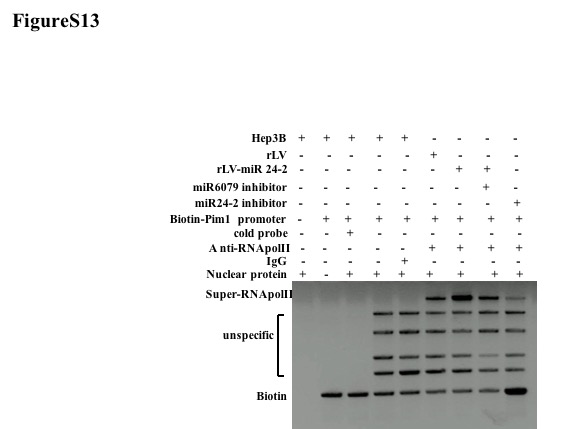
**

**
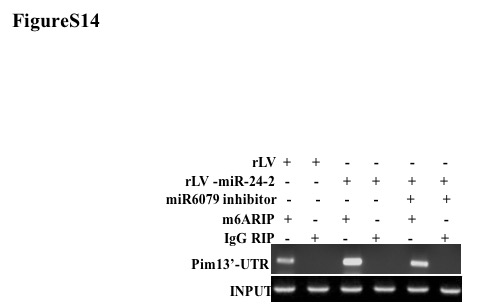
**

**
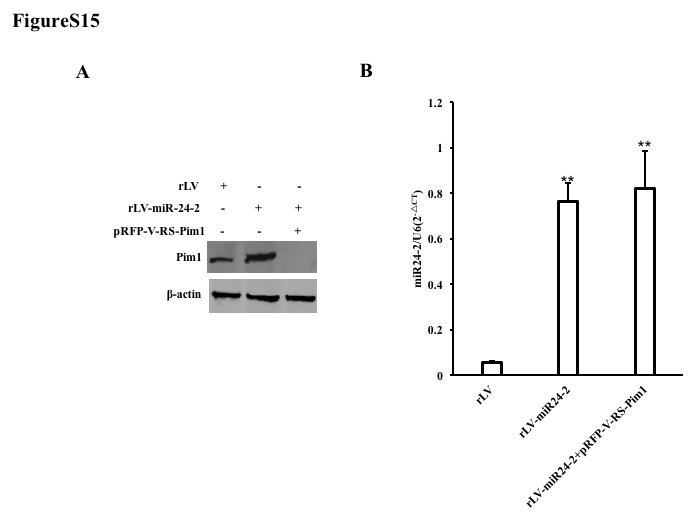
**

**
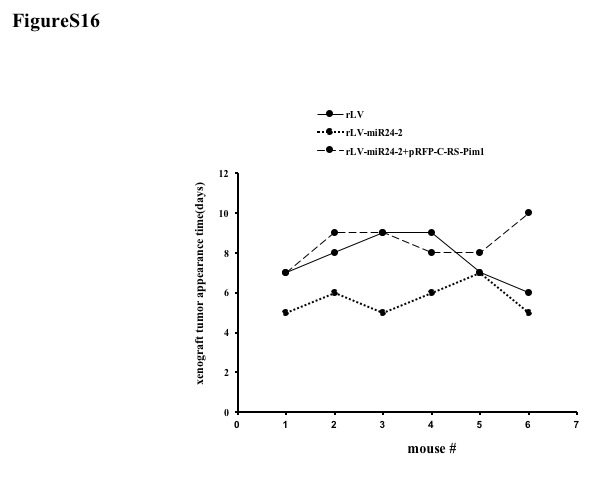
**

**
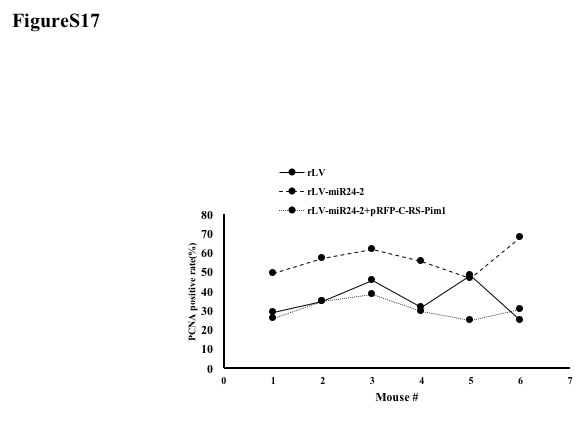
**

**Supplemental Figure legends**

**FigureS1** Expression analysis of miR24-2 in human primary liver cancer tissue by Northern-western blotting. liver cancer tissue(***C***) and its para-cancerous liver tissues(***P***). U6 as internal control.

**FigureS2** The transcriptional analysis of Pim1 in human primary liver cancer tissue by RT-PCR. liver cancer tissue(***C***) and its para-cancerous liver tissues(***P***). β-actin as internal control.

**FigureS3** The transcriptional analysis of Src in human primary liver cancer tissue by RT-PCR and β-actin as internal control(A),Western blotting with anti-Src and β-actin as internal control.(B), anti-Src Immunohistochemical staining(Ca&b) in liver cancer tissue(***C***) and its para-cancerous liver tissues(***P***).

**FigureS4** The establishing of miR24-2 overexpressing stable cell lines. **A.** the map of pLVX-Zs-Green plasmid. **B.** the sequencing of pLVX-Zs-Green-miR24-2.

**FigureS5** The RT-PCR analysis for pre-miR24-2 in the two stable Hep3B cell lines by infecting with rLV or rLV–miR24-2 respectively**.** β-actin as internal control.

**FigureS6** The assay of pre-miR24-2 looping in overexpressing stable cell lines. **A.** The RT-PCR analysis for pre-miR24-2 looping using the outward-facing primers in the two stable Hep3B cell lines by infecting with rLV or rLV–miR24-2 respectively**.** β-actin as internal control. **B.** the mode of pre-miR24-2 loop structure.

**FigureS7** The METTL3 promoter luciferase assay in Hep3B cells infected with rLV-miR24-2 or rLV. Each value was presented as mean ± standard error of the mean (SEM) (Student’s t-test). Bar ± SEM. **, P<0.01; *, P<0.05.

**FigureS8** The METTL3 3’-UTR luciferase assay in Hep3B cells infected with rLV-miR24-2 or rLV. Each value was presented as mean ± standard error of the mean (SEM) (Student’s t-test). Bar ± SEM. **, P<0.01; *, P<0.05.

**FigureS9** Co-Immunoprecipitation(IP) with anti-JMJD2A followed by Western blotting with anti-HistoneH3 in rLV group , rLV-6079 group and rLV-6079 group plus pGFP- group ,respectively.

**FigureS10 A.** Western blotting with anti-JMJD2A in Hep3B infected with rLV, rLV-miR24-2, respectively. **B.** The real-time RT-PCR analysis for mature miR24-2 in the two stable Hep3B cell lines by infecting with rLV or rLV–24-2, respectively. U6 as internal control. Each value was presented as mean ± standard error of the mean (SEM) (Student’s t-test). Bar ± SEM. **, P<0.01; *, P<0.05.

**FigureS11 A.** Western blotting with anti-JMJD2A in Hep3B infected with rLV, rLV-miR24-2, rLV-miR24-2 plus miR6079 inhibitor ,respectively. **B.** The real-time RT-PCR analysis for mature miR24-2 in Hep3B infected with rLV, rLV-miR24-2, rLV-miR24-2 plus miR6079 inhibitor , respectively. U6 as internal control. Each value was presented as mean ± standard error of the mean (SEM) (Student’s t-test). Bar ± SEM. **, P<0.01; *, P<0.05. **C.** The real-time RT-PCR analysis for mature miR6079 in Hep3B infected with rLV, rLV-miR24-2, rLV-miR24-2 plus miR6079 inhibitor ,respectively. U6 as internal control. Each value was presented as mean ± standard error of the mean (SEM) (Student’s t-test). Bar ± SEM. **, P<0.01; *, P<0.05.

**FigureS12** Chromatin Immunoprecipitation(CHIP) with anti-H3K9me3 followed by PCR with Pim1 promoter primers. IgG CHIP as negative control. Pim1 promoter as INPUT. Western blotting with anti-RNA polII for each samples.

**FigureS13** Super-EMSA assay with anti-RNA polII and Biotin Pim1 promoter probe in Hep3B infected with rLV, rLV-miR24-2, rLV-miR24-2 plus miRmiR6079 inhibitor ,miR24-2 inhibitor, respectively.

**FigureS14** RNA Immunoprecipitation(RIP) with anti-M6A followed by RT-PCR with Pim1 3’UTR primers.

**FigureS15 A.** Western blotting with anti-JMJD2A in Hep3B infected with rLV, rLV-miR24-2, rLV-miR24-2 plus pGFP-V-RS-Pim1 ,respectively. **B.** The real-time RT-PCR analysis for mature miR24-2 in Hep3B infected with rLV, rLV-miR24-2, rLV-miR24-2 plus pGFP-V-RS-Pim1 , respectively. U6 as internal control. Each value was presented as mean ± standard error of the mean (SEM) (Student’s t-test). Bar ± SEM. **, P<0.01; *, P<0.05.

**FigureS16** The xenograft tumor appearance time in rLV group, rLV-miR24-2 group, rLV-miR24-2 plus pGFP-V-RS-Pim1 group, respectively. Each value was presented as mean ± standard error of the mean (SEM) (Student’s t-test). Bar ± SEM. **, P<0.01; *, P<0.05.

**FigureS17** The PCNA positive rate in xenograft tumor from rLV group, rLV-miR24-2 group, rLV-miR24-2 plus pGFP-V-RS-Pim1 group, respectively. Each value was presented as mean ± standard error of the mean (SEM) (Student’s t-test). Bar ± SEM. **, P<0.01; *, P<0.05
